# Metabolic sensor *O*-GlcNAcylation regulates erythroid differentiation and globin production via BCL11A

**DOI:** 10.1186/s13287-022-02954-5

**Published:** 2022-06-23

**Authors:** Sudjit Luanpitpong, Xing Kang, Montira Janan, Kanjana Thumanu, Jingting Li, Pakpoom Kheolamai, Surapol Issaragrisil

**Affiliations:** 1 Siriraj Center of Excellence for Stem Cell Research, Faculty of Medicine Siriraj Hospital, Mahidol University, 2 Siriraj Hospital, Bangkoknoi, Bangkok, 10700 Thailand; 2 Synchrotron Light Research Institute (Public Organization), Nakhon Ratchasima, Thailand; 3 Institute of Precision Medicine, Department of Burns, The First Affiliated Hospital, Sun Yat-Sen University, Guangzhou, China; 4 Center of Excellence in Stem Cell Research and Innovation, Faculty of Medicine, Thammasat University, Pathum Thani, 12120 Thailand; 5 Division of Hematology, Department of Medicine, Faculty of Medicine Siriraj Hospital, Mahidol University, Bangkok, Thailand

**Keywords:** *O*-GlcNAcylation, Hematopoietic stem cells, Erythroblasts, Erythropoiesis, Erythroid differentiation, Erythroid maturation, Enucleation, Globin production

## Abstract

**Background:**

Human erythropoiesis is a tightly regulated, multistep process encompassing the differentiation of hematopoietic stem cells (HSCs) toward mature erythrocytes. Cellular metabolism is an important regulator of cell fate determination during the differentiation of HSCs. However, how *O*-GlcNAcylation, a posttranslational modification of proteins that is an ideal metabolic sensor, contributes to the commitment of HSCs to the erythroid lineage and to the terminal erythroid differentiation has not been addressed.

**Methods:**

Cellular *O*-GlcNAcylation was manipulated using small molecule inhibition or CRISPR/Cas9 manipulation of catalyzing enzyme *O*-GlcNAc transferase (OGT) and removing enzyme *O*-GlcNAcase (OGA) in two cell models of erythroid differentiation, starting from: (i) human umbilical cord blood-derived CD34^+^ hematopoietic stem/progenitor cells (HSPCs) to investigate the erythroid lineage specification and differentiation; and (ii) human-derived erythroblastic leukemia K562 cells to investigate the terminal differentiation. The functional and regulatory roles of *O*-GlcNAcylation in erythroid differentiation, maturation, and globin production were investigated, and downstream signaling was delineated.

**Results:**

First, we observed that two-step inhibition of OGT and OGA, which were established from the observed dynamics of *O*-GlcNAc level along the course of differentiation, promotes HSPCs toward erythroid differentiation and enucleation, in agreement with an upregulation of a multitude of erythroid-associated genes. Further studies in the efficient K562 model of erythroid differentiation confirmed that OGA inhibition and subsequent hyper-*O*-GlcNAcylation enhance terminal erythroid differentiation and affect globin production. Mechanistically, we found that BCL11A is a key mediator of *O*-GlcNAc-driven erythroid differentiation and β- and α-globin production herein. Additionally, analysis of biochemical contents using synchrotron-based Fourier transform infrared (FTIR) spectroscopy showed unique metabolic fingerprints upon OGA inhibition during erythroid differentiation, supporting that metabolic reprogramming plays a part in this process.

**Conclusions:**

The evidence presented here demonstrated the novel regulatory role of *O*-GlcNAc/BCL11A axis in erythroid differentiation, maturation, and globin production that could be important in understanding erythropoiesis and hematologic disorders whose etiology is related to impaired erythroid differentiation and hemoglobinopathies. Our findings may lay the groundwork for future clinical applications toward an ex vivo production of functional human reticulocytes for transfusion from renewable cell sources, i.e., HSPCs and pluripotent stem cells.

**Supplementary Information:**

The online version contains supplementary material available at 10.1186/s13287-022-02954-5.

## Background

Blood transfusion is essential for millions of patients with medical conditions as diverse as hereditary blood disorders, e.g., thalassemia and sickle cell anemia, renal failure, trauma, and cancers [1]. However, challenges associated with the use of donated blood include the risks of transmitted viral infections, such as HIV and hepatitis B, and transfusion reactions from incompatible minor blood group antigens, particularly in those who receive serial transfusion [2, 3]. Together with the concern regarding the shortage of blood supply globally [1, 4], numerous efforts are being made to develop functional reticulocytes in vitro from renewable sources, including human hematopoietic stem/progenitor cells (HSPCs), pluripotent stem cells, e.g., human embryonic stem cells (hESCs) and human-induced pluripotent stem cells (hiPSCs), and immortalized erythroblasts [4–7]. Currently, culture systems to generate erythroid cells in vitro have several limitations, including laborious process, limited cell number, and poor enucleation [6, 8]. hESC- and hiPSC-derived erythroid cells, in particular, primarily expressed fetal Ɣ-globin instead of adult β-globin [4, 9]. Thus, understanding the mechanisms that regulate erythropoiesis and globin production would aid in the development of an efficient in vitro culture system to replace transfusion banking in the future.

Cellular metabolism has recently been shown to play critical roles in cell fate determination during the differentiation of HSPCs [10, 11]. More specifically, a previous study reported that glucose and glutamine utilization toward nucleotide biosynthesis can dictate the erythroid commitment of HSPCs and that inhibition of glutamine would favor myelomonocytic fates [12]. *O*-GlcNAcylation is a nutrient-sensitive posttranslational modification involving two important enzymes, *O*-GlcNAc transferase (OGT) and *O*-GlcNAcase (OGA). The OGT uses UDP-GlcNAc, a product from hexosamine biosynthetic pathway (HBP), to add an N-acetylglucosamine (GlcNAc) moiety to the specific serine and/or threonine residues of target proteins, while the OGA removes the *O*-GlcNAc moiety [13, 14]. Because the HBP consumes glucose and glutamine, acetyl coenzyme A, and nucleotide uridine-5’-triphosphate (UTP), which are derived from amino acids, lipids, and nucleotide metabolism, the change in *O*-GlcNAc level generally reflects the altered cellular metabolism [13]. Interestingly, *O*-GlcNAcylation has been shown to modulate Ɣ-globin through the GATA1-FOG1-Mi2β repressor complex [15]. Moreover, the decreased *O*-GlcNAc level was detected in two erythroid cell models upon their induction toward terminal differentiation—the murine-derived erythroid progenitor G1E-ER4 cells, of which differentiation is mediated by GATA1, and the human-derived erythroblastic leukemia K562 cells, of which differentiation is mediated by sodium butyrate (NaB) [15, 16]. Prolonged treatment of the G1E-ER4 cells with thiamet G, an OGA inhibitor (OGAinh), for 14 days led to a slight increase in Ter-119^+^ erythroid cells, while it decreased hemoglobin-expressed cells by 10% when compared with its counterpart control. Recently, phosphoproteomics revealed dynamic changes in phosphorylation events coordinating terminal erythroid maturation, supporting the importance of posttranslational modifications in the process [17].

Human erythropoiesis is a tightly regulated, complex multistep process from the hematopoietic stem cells (HSCs) to the mature erythrocytes [18]; however, the roles of *O*-GlcNAcylation in erythropoiesis starting from the lineage specification of HSCs and during different stages of erythroblasts have not been addressed. In the present study, cellular *O-*GlcNAcylation was modified by using small molecule inhibition or CRISPR/Cas9 manipulation of OGA and OGT in human umbilical cord blood (UCB)-derived CD34^+^ HSPCs to evaluate its effect on lineage specification and erythroid maturation and in human-derived erythroblastic leukemia K562 cells that can be induced to undergo terminal differentiation with sufficient globin production. Following two-step inhibition of OGT and OGA in CD34^+^ HSPCs, the strategy of which developed from the cellular *O*-GlcNAc level during erythropoiesis, we observed the faster erythroid maturation and enucleation when compared with control. Induction of terminal differentiation and globin production upon OGA inhibition was validated in the efficient K562 model of erythroid differentiation. We further provide molecular insight into how *O*-GlcNAcylation controls erythroid maturation and globin production, that is via BCL11A. We believe that the novel insight gained from our study could be important in understanding erythropoiesis and may help improving the efficiency of ex vivo production of human reticulocytes from HSPCs or even hiPSCs for future clinical use.

## Methods

### Reagents

OGAinh thiamet G was obtained from Tocris Bioscience (Bristol, UK), while OGT inhibitors (OGTinh) alloxan and OSMI-1 were from Sigma-Aldrich (St. Louis, MO). Dimethyl sulfoxide (DMSO; Merck, Darmstadt, Germany) was used as a solvent for thiamet G and OSMI-1 to prepare 1000X stock solutions, and 0.1% DMSO was used as a vehicle (nontreated) control. Antibodies for globins were obtained from Santa Cruz Biotechnology (Dallas, Texas), while other antibodies were from Abcam (Cambridge, UK), unless otherwise mentioned. All cytokines were obtained from Miltenyi (Bergisch Gladbach, Germany). Full details of key resources can be found in Additional file 1: Table S1.

### Purification of CD34^+^ HSPCs

Human UCB was collected from healthy newborns at birth after informed consent was obtained from their mothers and after approval by the Siriraj Institutional Review Board (COA Nos. Si 564/2018 and Si 090/2020). Mononuclear cells were enriched from the UCB samples over a Ficoll-Paque gradient (GE Healthcare, Marlborough, MA), and CD34^+^ HSPCs were subsequently isolated using a CD34 MicroBead Kit and MS Column (Miltenyi) according to the manufacturer's protocol. The purity was approximately 90%, as evaluated by flow cytometry (FACSCalibur, BD Biosciences, San Jose, CA).

### Cell culture

Isolated CD34^+^ HSPCs were expanded for 5 days in Iscove’s Modified Dulbecco’s Medium (IMDM; Thermo Fisher Scientific, Waltham, MA) supplemented with 10% (v/v) fetal bovine serum (FBS) (PAN-Biotech, Aidenbach, Germany) and 25 ng/mL recombinant human (rh) SCF, rhIL-3, rhIL-6 and rhFLT3-L (Miltenyi) prior to experiments. Human-derived erythroblastic leukemia K562 cells were purchased from American Type Culture Collection (ATCC; Manassas, VA). The cells were cultured in IMDM supplemented with 10% (v/v) FBS, 100 U/mL penicillin, and 100 mg/mL streptomycin. The medium was replaced every 3 days throughout the entire culture period, and the cells were passaged when their density reached 1 × 10^6^ cells/mL. All cells were maintained in a humidified atmosphere of 5% CO_2_ at 37 °C.

### CRISPR/Cas9-mediated gene manipulation

All-in-one lentiviral plasmids carrying *Streptococcus pyogenes* CRISPR/Cas9 (SpCas9), puromycin resistance gene, and single-guide RNA (sgRNA) sequences specific to human *OGT*, *MGEA5* (encoding OGA), and *BCL11A* genes were purchased from GenScript (Piscataway, NJ) The oligo sequences of all sgRNAs are listed in Additional file 1: Table S2. The plasmids were transfected into HEK293T packaging cells to produce lentiviral particles. The particles were transduced into K562 cells in the presence of 8 μg/mL hexadimethrine bromide for 48 h and were treated with 1 μg/mL puromycin for 1 month for the selection of stably transfected cells. After which, single-cell isolation by limiting dilution was performed to generate the single-cell derived clones. Downregulation of the target proteins was confirmed by Western blotting prior to the experiments.

### Genomic DNA sequencing and ICE analysis

Genomic DNA was isolated using a PureLink Genomic DNA Mini Kit (Invitrogen, Waltham, MA). The target regions for DNA sequencing were amplified by polymerase chain reaction (PCR) using Q5 High-Fidelity DNA Polymerase (New England Biolabs, Ipswich, MA) with specific primers, and the resulting PCR products were purified by a GenepHlow Gel/PCR kit (Geneaid, New Taipei City, Taiwan). A total of 0.2 μg PCR product was then used for DNA sequencing using ABI PRISM BigDye Terminator Cycle Sequencing Kit v3.1 (1st BASE, Singapore). The presence of insertion/deletion (INDEL) mutations in each target gene was determined by Inference of CRISPR Edits (ICE), the web-based analysis tool (available at https://ice.synthego.com/).

### Colony-forming unit (CFU) assay for human progenitors

CFU assay was performed using MethoCult H4435 Enriched (STEMCELL Technologies, Vancouver, Canada), which supports the growth of multipotential progenitor cells (CFU-granulocyte, erythrocyte, macrophage, megakaryocyte (CFU-GEMM)), granulocyte and macrophage progenitor cells (CFU-granulocyte, macrophage (CFU-GM)), and erythroid progenitor cells (burst-forming unit-erythroid (BFU-E) and CFU-erythroid (CFU-E)), according to the manufacturer’s instructions. Briefly, 500 starting cells were gently dispersed in enriched methylcellulose (MC) medium containing rhSCF, rhGM-CSF, rhIL-3, rhIL-6, rhG-CSF, and erythropoietin (rhEPO) and seeded onto 35-mm dishes or 6-well plates. Cells were cultured for 14 days, and colonies were identified and counted under an inverted microscope (Eclipse Ti-U with NiS-Elements, Nikon, Tokyo, Japan). Percentage of each progenitor cell colony over total number of colonies was calculated and plotted.

### Erythroid differentiation from CD34^+^ HSPCs and K562 cells

After expansion, CD34^+^ HSPCs were cultured in a three-stage erythroid culture system in the so-called basal erythroid differentiation medium: IMDM containing 3% (v/v) human AB serum, 2% (v/v) FBS, 10 μg/mL insulin, 3 U/mL heparin, 3 U/mL rhEPO (EPREX, Janssen-Cilag, Zug, Switzerland), and 200 μg/mL transferrin [1]. On days 0–8 of culture (first stage), the medium was additionally supplemented with 10 ng/mL rhSCF and 1 ng/mL rhIL-3, while on days 8–11 (second stage), it was additionally supplemented with 10 ng/mL rhSCF only. In the last stage, additional transferrin was supplemented to the medium to a final concentration of 500 μg/mL.

Induction of erythroid differentiation from K562 cells was modified from the procedure described previously by Uchida and colleagues [19]. K562 cells were maintained in IMDM supplemented with 10% (v/v) FBS and 1 μM imatinib, a BCR/ABL tyrosine kinase inhibitor, in T25 flask for 24 h. After which, the cells were cultured in EPO-based differentiation medium containing 20% FBS, 2 U/mL rhEPO, 10 ng/mL insulin, 0.5 mg/mL transferrin, and 2% (v/v) bovine serum albumin (BSA, Sigma-Aldrich), 2−10 μM hemin (iron-containing porphyrin), 20 ng/mL rapamycin, and 0.2 μM decitabine (5-Aza-2’-deoxycytidine) in IMDM for up to 14 days. Under certain conditions, erythroid maturation was additionally induced with 0.6 mM NaB [20]. The complete medium was changed every 2 days, and the cells were maintained at a density below 1 × 10^6^ cells/mL.

### Flow cytometry

Cultured erythroid cells were characterized by incubation with FITC-conjugated anti-human CD71 (transferrin receptor) and PerCP-Cy5.5- or APC-conjugated anti-human CD235a (glycophorin A, GPA) (BioLegend, San Diego, CA) for 15 min at room temperature and analyzed using FACSCalibur flow cytometer (BD Biosciences). Subsets of CD34^+^ HSPC-derived erythroid cells were characterized using CD71 and size (forward scatter, FSC) on the basis that mature cells are small and express low CD71. Fluorescence-activated cell sorting (FACS) enrichment was performed using a FACSAria cell sorter (BD Biosciences).

### Morphological analysis

A total of 1 − 3 × 10^4^ cells were harvested at the indicated time, immobilized onto a glass by cytospin centrifugation, and left to dry at 4 °C for 24 h. Slides were then stained with 2 mL Wright staining solution for 3 min, washed several times with an equal volume of distilled water and left to dry at room temperature. Cell morphology was then determined by light microscopy (Olympus CX31, Tokyo, Japan), and the percentage of cells in different stages was determined by scoring at least 300 cells.

### Western blot analysis

The cells were harvested and lysed by incubation with a lysis buffer (Cell Signaling Technology, Denvers, MA) containing protease inhibitors (Roche Diagnostics, Mannheim, Germany) at 4 °C for 30 min. Concentrations of the isolated protein were then determined by a BCA protein assay (Pierce Biotechnology). Approximately 10 − 100 μg was subjected to SDS-PAGE and transferred onto PVDF membranes. The membranes were blocked with 5% (w/v) skim milk, incubated with appropriate primary antibodies at 4 °C overnight, and subsequently incubated with HRP-conjugated secondary antibodies for 1 h at room temperature. An enhanced chemiluminescence detection system (Merck Millipore, Burlington, MA) was then used to detect the antibody-bound proteins on a digital imager ImageQuant LAS (GE Healthcare). Blots were reprobed with anti-β-actin antibody to establish loading control. For quantitative analysis, immunoblot signals were quantified by densitometry, normalized to the loading control, and calculated as the fold difference relative to control.

### RNA isolation and quantitative real-time PCR (qPCR) analysis

Total RNA was isolated from cells using TRIzol reagent (Invitrogen), and cDNA was synthesized using a RevertAid First Strand cDNA Synthesis Kit (Thermo Fisher Scientific) and oligo (dT) primers (Invitrogen). qPCR analysis was performed on a 7500 Fast real-time PCR using a Power SYBR Green PCR Master Mix (Applied Biosystems, Waltham, MA). The PCR reaction consisted of 1X SYBR Green master mix, 200 nM of forward and reverse primers (Additional file 1: Table S3), and 1 μL of template cDNA. Initial enzyme activation was performed at 95 °C for 10 min, followed by 40 cycles of denaturation at 95 °C for 15 s and primer annealing/extension at 60 °C for 1 min. The mRNA level of each gene was normalized against the mRNA level of the housekeeping gene *GAPDH*.

### Fourier transform infrared (FTIR) sample preparation and microspectroscopy analysis

A drop of 5 μL of 3 × 10^5^ cells was deposited onto IR-transparent 2-mm-thick barium fluoride windows and air-dried in a desiccator overnight. The slides were then washed with distilled water five times to eradicate a saline crystal, air-dried, and stored in a desiccator until spectra were acquired. The spectra were acquired at BL4.1 IR Spectroscopy and Imaging Beamline at the Synchrotron Light Research Institute with a Vertex 70 FTIR Spectrometer (Bruker Optics, Ettlingen, Germany) coupled with an IR microscope (Hyperion 2000, Bruker Optics) and a mercury-cadmium-telluride (MCT) detector cooled with liquid nitrogen over the measurement range from 4000 to 800 cm^−1^, as previously described [21]. The microscope was connected to a software-controlled microscope stage and placed in a specially designed box that was purged by dry air. The measurements were performed using an aperture size of 10 × 10 μm with a spectral resolution of 4 cm^−1^, with 64 scans co-added. Spectral acquisition and instrument control were performed using OPUS 7.2 software (Bruker Optics). Spectra from each sample group were analyzed by using principal component analysis (PCA). Data were preprocessed by performing a baseline correction and then normalized using extended multiplicative signal correction using the spectral regions from 3000 to 2800 cm^−1^ and 1800 to 900 cm^−1^ using Unscrambler 10.1 software (CAMO, Oslo, Norway).

### Statistical analysis

The data represent means ± SD from three or more independent experiments. Statistical analysis was performed using two-sided Student's *t* test or one-way ANOVA followed by a Tukey’s multiple comparisons test. *P* < 0.05 was considered statistically significant.

## Results

### Inhibition of OGT promoted the differentiation of HSPCs into erythroid progenitor BFU-E

**Fig. 1:**
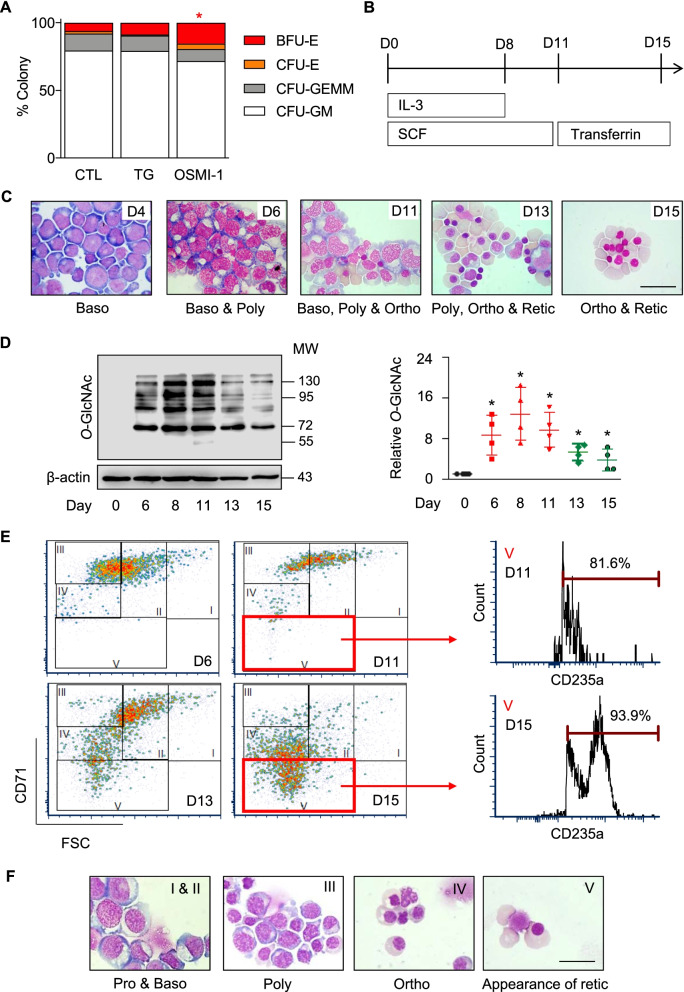
Characterization of erythroblasts differentiated from UCB-derived CD34^+^ HSPCs. **A** Inhibition of OGT by OSMI-1 promoted the differentiation of CD34^+^ HSPCs into the more committed erythroid progenitors BFU-E as evaluated by CFU assay. Colonies were visualized and scored under an inverted microscope at 14 days of culture. Data are mean (*n* = 3). ^*^P < 0.05 versus % BFU-E colony of nontreated control (CTL); two-sided Student’s *t* test. TG: thiamet G. **B** Schematic diagram of erythroid differentiation from CD34^+^ HSPCs in the three-stage erythroid culture system. **C** Representative morphology of differentiated cells on Wright-stained cytospin slides on various days of culture (days 4–15) showing different stages of erythroid differentiation, including basophilic normoblasts (Baso), polychromatic normoblasts (Poly), orthochromatic erythroblasts (Ortho), and reticulocytes (Retic). Scale bar = 20 μm. **D** (left) Western blot analysis of *O*-GlcNAc level along the time course of erythroid differentiation using anti-*O*-GlcNAc-specific antibody (RL2). Blots were reprobed with anti-β-actin antibody to establish a loading control (see also Additional file 2: Fig. S2 for the OGA and OGT levels). (right) Quantitative analysis of *O*-GlcNAc level by densitometry after normalization to the loading control is shown. Data are mean ± SD (*n* = 4). ^*^P < 0.05 versus cells at the start of culture (day 0); two-sided Student's *t* test. **E** Different subsets of differentiated erythroid cells, stages I–V, based on their maturity using CD71 versus FSC together with CD235a (see also Additional file 2: Fig. S4 for the gating strategy). Stage V cells were confirmed to express CD235a. **F** Representative morphology of sorted stage I–V cells on Wright-stained cytospin slides. Scale bar = 20 μm

To first assess the potential role of *O*-GlcNAcylation in hematopoietic differentiation, we modulated the intracellular *O*-GlcNAc level in CD34^+^ HSPCs using OGAinh thiamet G and OGTinh OSMI-1 and performed CFU assay to evaluate hematopoietic cells at different stages of differentiation, from HSPCs to lineage-restricted progenitor cells. Figure 1A shows that OSMI-1 significantly promoted the differentiation of HSPCs into erythroid progenitor BFU-E (*P* < 0.05) but had a minimal effect on the differentiation of HSPCs into other progenitor cells, including CFU-E, CFU-GM, and CFU-GEMM (see also Additional file 1: Table S4 and Additional file 2: Fig. S1), thus hinting about the role of *O*-GlcNAcylation in erythroid differentiation.

### Changes in cellular *O*-GlcNAcylation during different stages of erythroid differentiation

The established three-stage erythroid culture system was used to induce CD34^+^ HSPCs toward erythroid differentiation, as schematically depicted in Fig. 1B. During the course of differentiation, the HSPCs gradually converted to cells with morphology similar to those of pronormoblasts (not shown), basophilic normoblasts, polychromatic normoblasts, orthochromatic erythroblasts, and reticulocytes (Fig. 1C), recapitulating the normal sequence of maturation [22]. Interestingly, the cellular *O*-GlcNAc level, as evaluated by Western blotting using a specific anti-*O*-GlcNAc antibody (RL2), remarkably increased on days 6–11 of culture and gradually decreased at the last stage of culture (day 11 onwards) (Fig. 1D). It is worth noting here that the precise mechanism of how cellular *O*-GlcNAcylation changes during erythroid differentiation remains unclear and is likely to be complex and diverse since *O*-GlcNAcylation senses and responds to a wide variety of stimuli and stresses. However, our results indicate that this might involve a modulation of *O*-GlcNAc cycling enzymes, as a striking increase in both OGT and OGA at protein and gene levels was observed in the late stage of differentiation (Additional file 2: Fig. S2).

Immunophenotypic kinetics of erythroid markers CD71 and CD235a [23, 24] revealed that the differentiated cells mostly expressed CD71 in an early phase. Upon maturation, the expression of CD71 substantially decreased as opposed to an observed increase in CD235a (Additional file 2: Fig. S3). To extensively elaborate the effect of *O*-GlcNAcylation on erythroid differentiation, we characterized CD34^+^ HSPC-derived erythroid cells into different subsets, hereafter termed stages I–V, based on their maturity using CD71 and size (FSC) on the basis that mature cells are small and express low CD71 [25, 26] (see also Additional file 2: Fig. S4 for gating strategy). The most mature, stage V subset was CD71^low^FSC^low^CD235a^high^ (Fig. 1E and Additional file 2: Fig. S5). FACS sorting and subsequent Wright staining of cytospinned cells in the stages I–V confirmed that the adopted flow cytometric analysis could clearly distinguish the stages of erythroid development (Fig. 1F).

### Two-step inhibition of OGT and OGA promotes erythroid differentiation and maturation

**Fig. 2:**
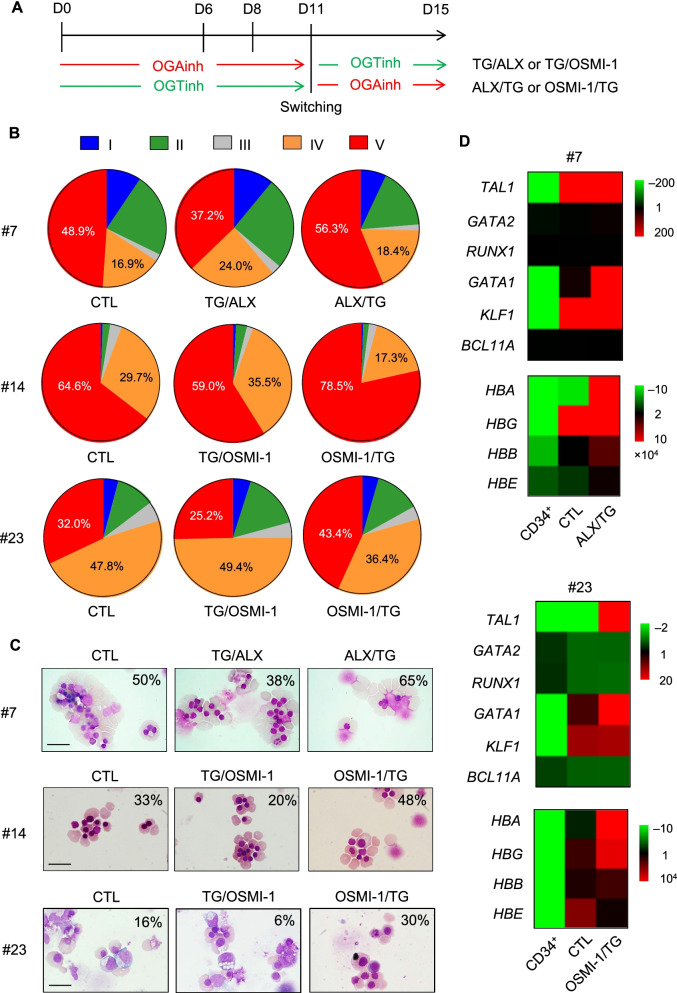
Two-step inhibition of OGT and OGA induces erythroid differentiation and enucleation. **A** Schematic diagram of the treatment strategy developed based on the cellular *O*-GlcNAc level during erythroid differentiation from CD34^+^ HSPCs. Treatment was either OGAinh/OGTinh or OGTinh/OGAinh, replacing every 2 days and switching on day 11, for a total of 15 days—alloxan (ALX) was used at 1 mM, thiamet G (TG) was at 10 μM, and OSMI-1 was at 5 μM. **B** Percentages of differentiated erythroblasts in stages I–V as analyzed by flow cytometry using FSC versus CD71, derived from HSPCs of donors #7, #14, and #23 on day 15 of culture. **C** Representative morphology of treated cells on Wright-stained cytospin slides on day 15 of culture. Percentage of enucleated cells from at least 300 cells is indicated on the micrograph. Scale bar = 20 μm. **D** qPCR of genes involved in erythroid development and function in the differentiated cells derived from CD34^+^ HSPCs of donors #7 and #23 on day 15 of culture. Data were normalized to *GAPDH* and represented in a heatmap

Cellular *O*-GlcNAcylation can be reversibly manipulated via small molecule inhibition of OGA, using thiamet G, and of OGT using alloxan and OSMI-1. Having demonstrated the dynamics of *O*-GlcNAc level during erythroid differentiation with a higher level on days 6–11 of culture (Fig. 1D), we divided the treatments into two phases—phase 1 from day 0 to day 11 and phase 2 from day 11 onwards. Treatments were switched between OGAinh and OGTinh on day 11, as schematically depicted in Fig. 2A. CD34^+^ HSPCs were obtained from various healthy newborns (cases #7, #14, and #23; Additional file 1: Table S5) and were treated with either OGAinh/OGTinh or OGTinh/OGAinh for a total of 15 days, or left untreated (control), and the percentages of erythroblasts in stages I–V were analyzed by flow cytometry using CD71 versus FSC and the gating strategy described in Fig. 1E. Treatment of the cells with OGTinh, either alloxan (#7) or OSMI-1 (#14 and #23), for 11 days, followed by OGAinh, that is, thiamet G, for 4 days substantially induced erythroid differentiation as evidenced by an increase in the percentage of cells in stage V, consisting of orthochromatic erythroblasts and reticulocytes, in all cases (Fig. 2B) (see also Additional file 2: Fig. S6 for statistical analysis). Consistently, morphological appearances of OGTinh/OGAinh-treated cells by Wright staining exhibited more mature erythroblast phenotypes and greater enucleation (Fig. 2C).

To substantiate that OGTinh/OGAinh treatment promoted CD34^+^ HSPCs toward erythroid differentiation, we conducted qPCR to evaluate mRNA expression of key hematopoietic transcription factors, including *TAL1*, *GATA2*, and *RUNX1* [27, 28], and erythroid master regulators, including *GATA1* and *KLF1* [29, 30]. Gene expression levels normalized to the housekeeping *GAPDH* comparing CD34^+^ HSPCs, nontreated control, and OGTinh/OGAinh-treated cells were represented in a heatmap. Upon erythroid differentiation for 15 days, a striking upregulation of *TAL1*, *GATA1*, and *KLF1* was observed when compared to CD34^+^ HSPCs at the start of culture (Fig. 2D). Treatment of the cells with OGTinh/OGAinh further upregulated *TAL1* and *GATA1* when compared to control cells on the same day. We also evaluated the Ɣ-globin repressor *BCL11A* [31] along with various hemoglobin genes, including *HBA*, *HBG*, *HBB*, and *HBE*, at 15 days after differentiation*.* As expected, the expression of all globin genes, particularly *HBA*, was extremely high in the differentiated erythroid cells. Interestingly, upregulation of *HBA* by approximately two-fold was detected in the presence of OGTinh/OGAinh when compared to control cells, while other globin genes were minimally changed. These data support the involvement of *O*-GlcNAcylation in both erythropoiesis and hemoglobin production.

### Changes in cellular *O*-GlcNAcylation in the efficient K562 model of erythroid differentiation

**Fig. 3:**
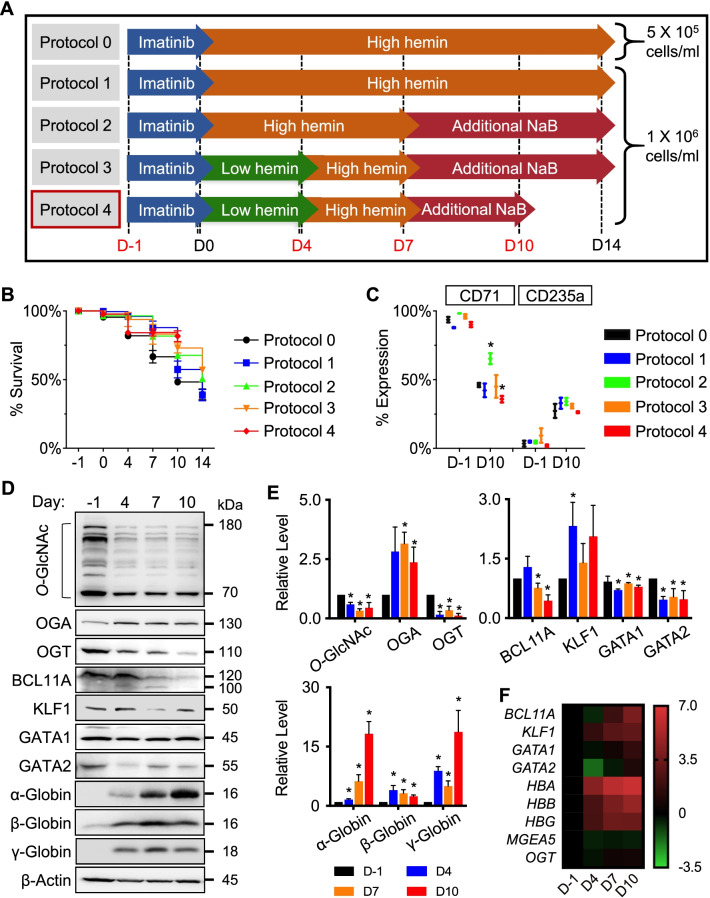
Optimization of the culture protocol for efficient erythroid differentiation from human-derived erythroblastic K562 cells. **A** Schematic diagram describing the original (Protocol 0) and the modified protocols (Protocols 1−4) for the induction of erythroid differentiation using EPO-based medium in K562 cells. (**B**,** C**) Cell viability **B** and the percentages of CD71^+^ and CD235a^+^ cells **C** at various times of culture from different protocols. Data are mean ± SD (*n* = 3). ^*^P < 0.05 versus Protocol 0 on the same day of culture; two-sided Student's *t* test. **D** Western blot analysis of cellular *O*-GlcNAcylation and its cycling enzymes, erythroid-associated proteins, and globin proteins along the course of differentiation (day −1 to day 10). Blots were reprobed with anti-β-actin antibody to establish a loading control. **E** Quantitative analysis of *O*-GlcNAc, OGA, OGT (upper, left), erythroid-associated proteins (upper, right), and globin proteins (lower) by densitometry after normalization to the loading control is shown. Data are mean ± SD (*n* = 3). ^*^P < 0.05 versus cells at the start of culture (day −1); two-sided Student's *t* test. **F** qPCR of genes encoding key proteins in **D**. Data were normalized to *GAPDH* and represented in a heatmap

Due to limitations in cell number and case-to-case variations of CD34^+^-derived erythroid cells, which were likely the influence of maternal and neonatal factors, e.g., maternal age, gestational age, birth order, birth weight, infant sex, and length of umbilical cord [32, 33], human-derived erythroblastic leukemia K562 cells were used to further investigate the regulatory mechanisms behind terminal erythroid differentiation, which theoretically starts from proerythroblasts that proliferate and differentiate to generate reticulocytes [22]. We first optimized the differentiation protocol to obtain healthy cells in all stages and a sufficient amount of hemoglobin for protein analysis by modifying the cell density, concentrations, and duration of hemin used by Uchida et al. (Protocol 0) [19] as well as by employing NaB to induce erythroid maturation and enucleation, as schematically depicted in Fig. 3A. Cell viability and the percentages of cells that expressed early erythroid marker CD71 and late erythroid marker CD235a were determined at various times of culture. Figure 3B and C shows that while all five protocols (Protocols 0–4) generated similar percentages of CD71^+^ and CD235a^+^ cells at the end of culture, Protocol 4 yielded the highest viable cells (~ 80% versus ~ 50% in Protocol 0 on day 10) (Additional file 2: Fig. S7); thus, it was selected for further experiments.

We next profiled the cellular *O*-GlcNAcylation and its cycling enzymes OGA and OGT along with various key proteins associated with erythroid differentiation, including BCL11A, KLF1, GATA1, GATA2, and globin proteins upon erythroid differentiation by the optimized protocol using Western blotting. Figure 3D shows a significant decrease in *O*-GlcNAc level along the course of differentiation, associated with an increase in OGA and an opposing decrease in OGT levels and supporting the involvement of *O*-GlcNAcylation in the process. Consistent with previous studies reporting that GATA2 and BCL11A expressed at high level in early erythroid progenitors and declined in the later stages [29, 34], a decrease in GATA2 and BCL11A levels was similarly observed toward the end of culture in our settings (Fig. 3D and E). Production of α-globin, β-globin, and Ɣ-globin, as evaluated by Western blotting, was apparent after 4 days of differentiation, and the red color of cell pellets was noticeable (Additional file 2: Fig. S8). qPCR of genes encoding the aforementioned key erythroid proteins is shown in Fig. 3F. It is noteworthy that the remarkable decrease in BCL11A level on day 10, in opposed to *BCL11A* upregulation, suggested its regulation at the posttranslational level.

### Modulation of *O*-GlcNAc level via genetic inhibition of OGA and OGT in K562 cells

**Fig. 4:**
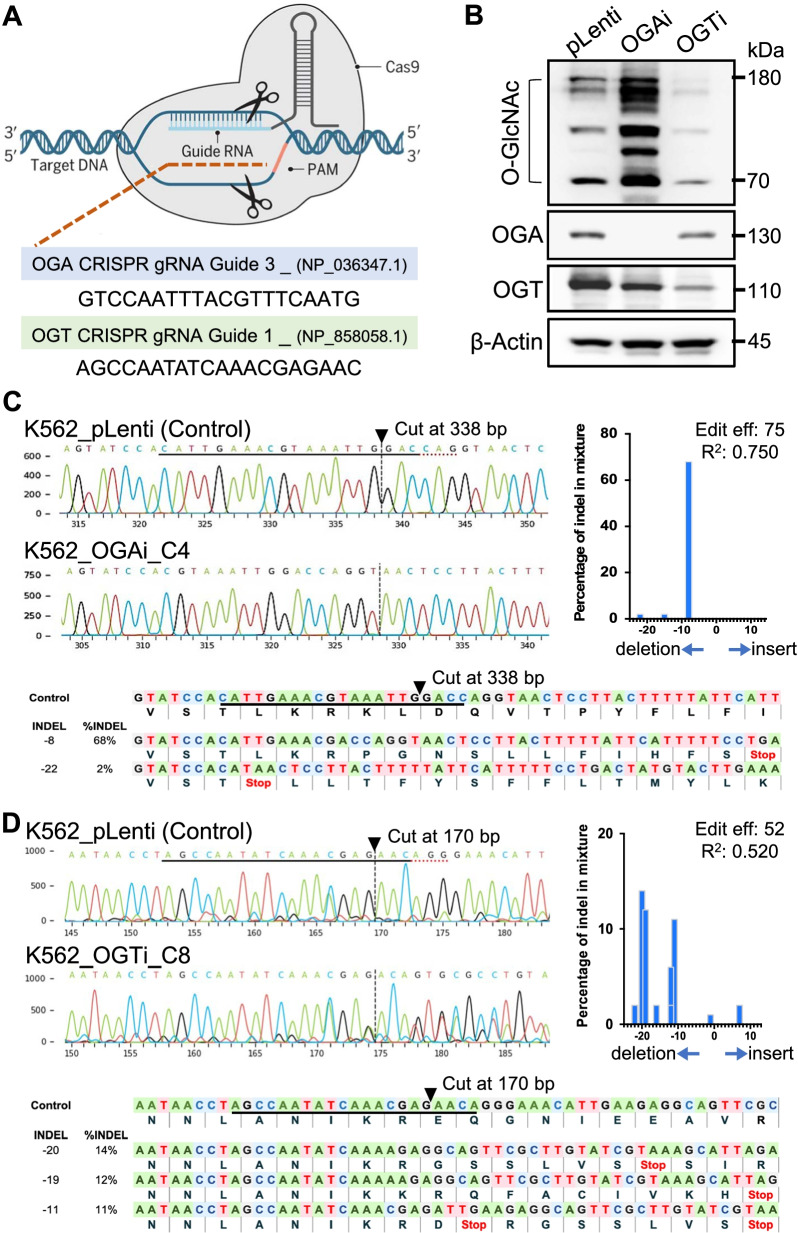
Inhibition of OGT and OGA by the CRISPR/Cas9 system and their effects on cellular *O*-GlcNAcylation in K562 cells. **A** Schematic illustration of CRISPR/Cas9-mediated repression of *MGEA5* (encoding OGA) and *OGT*. The oligo sequences of sgRNAs that specifically recognized the target DNA region are shown. **B** Western blot analysis of OGA, OGT, and *O*-GlcNAc levels showing knockdown efficiency and manipulation of *O*-GlcNAcylation in OGAi and OGTi cells in comparison with CRISPR control (pLenti) cells. Blots were reprobed with anti-β-actin antibody to establish a loading control. (**C**, **D**) (left) DNA sequencing spanning over the Cas9 cut site on *MGEA5* (**C**) and *OGT* (**D**) from the CRISPR control (pLenti) cells in comparison with the OGAi (**C**) and OGTi **D** cells. The guide sequence is underlined in black, and the PAM sequence is denoted by a dotted red underline in the control sample. Vertical dotted lines denote the expected cut site. (right) INDEL spectrum and ICE editing efficiency (Edit eff) determined by ICE software. (lower) The inferred sequences of *MGEA5* present in OGAi **C** and of *OGT* present in OGTi **D** cells and their relative proportions (%INDEL) were listed along with the translated amino acids

To determine the direct relationship between the cellular *O*-GlcNAc level and terminal erythroid differentiation, we used the CRISPR/Cas9 system to repress *MGEA5* (encoding OGA) and *OGT* in K562 cells, as schematically depicted in Fig. 4A. The cells were transduced with lentiviral particles comprising sgRNA against *MGEA5* (OGAi) or *OGT* (OGTi) and Cas9, subjected to single-clone selection, and their effect on OGT, OGA, and *O*-GlcNAc levels was determined by Western blotting. Figure 4B shows that the OGA level was abolished entirely in OGAi cells and the *O*-GlcNAc level was greatly increased when compared to CRISPR control (pLenti) cells and that although the OGT level was not completely abolished in OGTi cells, its *O*-GlcNAc level was significantly diminished. Subsequent genomic DNA sequencing confirmed the presence of INDEL mutations in the coding sequences of *OGA* and *OGT* in OGAi and OGTi K562 cells, with the ICE editing efficiencies of 75% and 52%, respectively (Fig. 4C and D). All major frameshift mutations in OGAi and OGTi cells generated early stop codons. Altogether, these data indicate that the selected clones of OGAi and OGTi cells can be used to ascertain the roles of *O*-GlcNAcylation in terminal erythroid differentiation in K562 cells.

### Hyper-*O*-GlcNAcylation via OGA inhibition enhances terminal erythroid differentiation

**Fig. 5:**
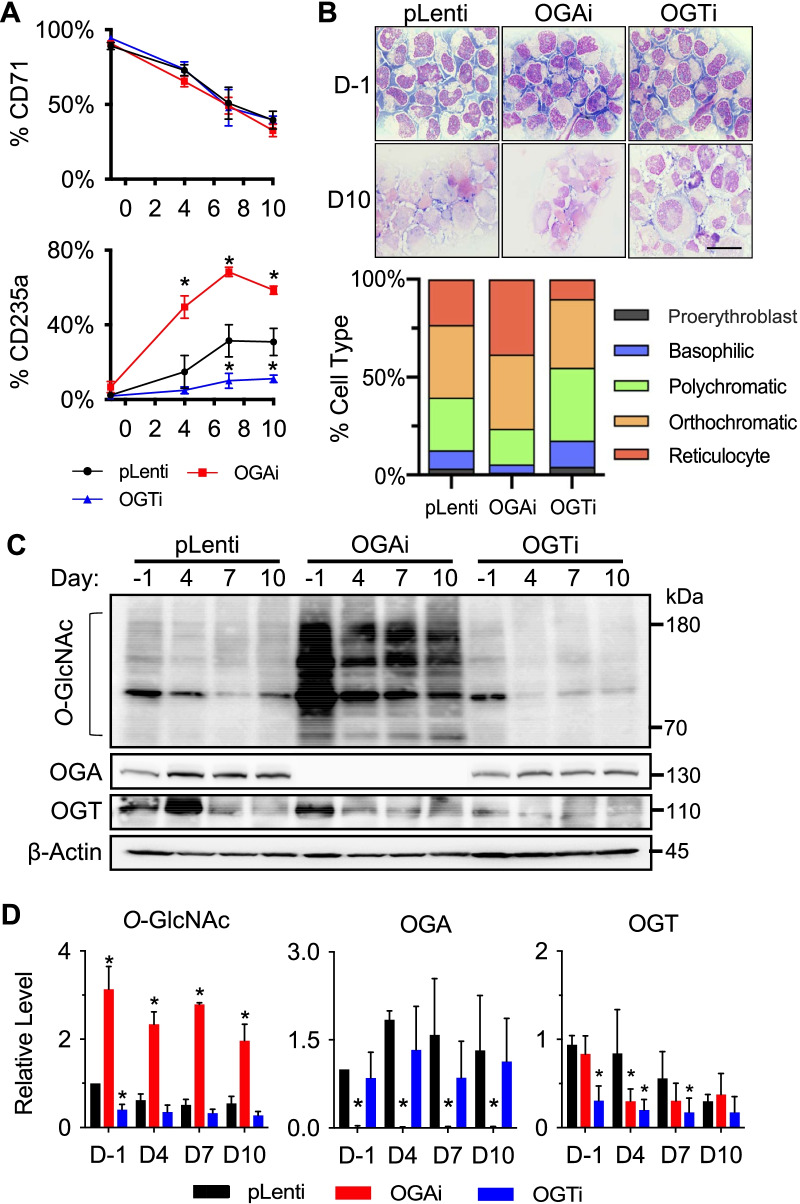
Inhibition of OGA induces erythroid differentiation in K562 cells. **A** Percentages of CD71^+^ and CD235a^+^ cells in OGAi, OGTi, and control (pLenti) cells at various times of culture in the optimized EPO-based medium. **B** Representative morphology of differentiated cells on Wright-stained cytospin slides before imatinib pre-exposure (day −1) and after 10 days of culture in EPO-based medium (upper) and its scoring for percentages of cells in different stages from at least 300 cells (lower). Scale bar = 30 μm. **C** Western blot analysis of cellular *O*-GlcNAc, OGA, and OGT levels upon terminal erythroid differentiation at various times (day −1 to day 10). Blots were reprobed with anti-β-actin antibody to establish a loading control. **D** Quantitative analysis of *O*-GlcNAc, OGA, and OGT levels by densitometry after normalization to the loading control is shown. Data are mean ± SD (*n* = 3). ^*^P < 0.05 versus pLenti cells on the same day of culture; two-sided Student's *t* test

OGAi, OGTi, and CRISPR control (pLenti) K562 cells were pushed toward erythroid differentiation using the established Protocol 4, and erythroid surface markers, cell morphology, and the expression of genes and proteins associated with erythroid differentiation were compared among them at various time points, e.g., before imatinib pre-exposure (day − 1) and after 4, 7, and 10 days of culture in EPO-based medium. While the percentage of cells expressing early erythroid marker CD71 was similar in all groups, the percentage of cells expressing late erythroid marker CD235a was found to be substantially increased in OGAi cells when compared to pLenti cells from day 4 onwards (Fig. 5A), consistent with the results from morphological analysis showing a greater percentage of reticulocytes in OGAi cells (Fig. 5B). On the contrary, the percentage of CD235a^+^ cells and reticulocytes was lowest in OGTi cells with the lowest *O*-GlcNAc level, thus supporting that the increased cellular *O*-GlcNAcylation (Fig. 5C and D) enhances terminal erythroid differentiation. Notably, the data obtained from the K562 model of erythroid differentiation were in agreement with the data from the last stage of three-stage erythroid differentiation from CD34^+^ HSPCs reporting the promoting role of OGA inhibition in the acquisition of orthochromatic erythroblasts and reticulocytes, indicating that OGA inhibition is particularly important in erythroid maturation and enucleation.

**Fig. 6:**
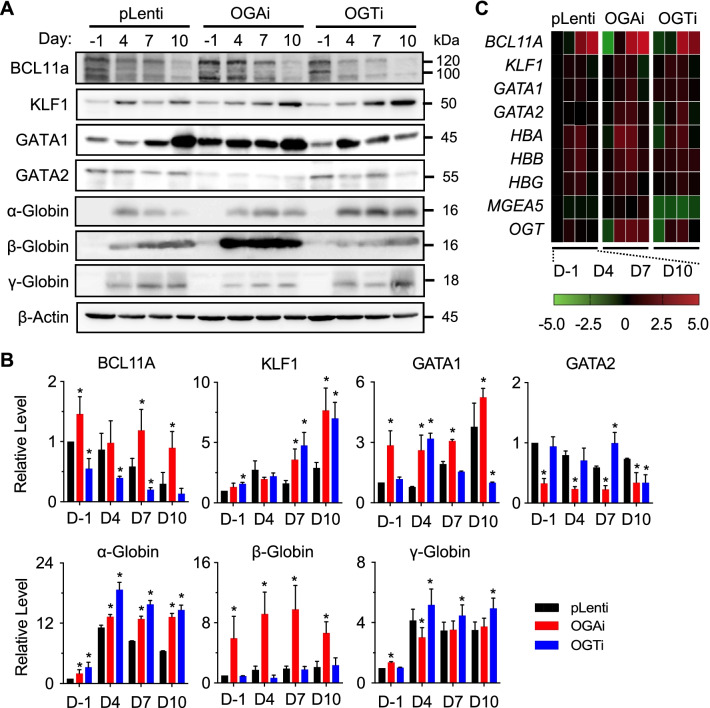
Inhibition of OGA and OGT affects erythroid-associated proteins and globin production in K562 cells. **A** Western blot analysis of erythroid-associated proteins, including BCL11A, KLF1, GAT1, and GATA2, and globin proteins, including α-, β-, and Ɣ-globin, in OGAi, OGTi, and control (pLenti) cells upon erythroid differentiation in EPO-based medium at various times (day −1 to day 10). Blots were reprobed with anti-β-actin antibody to establish a loading control. **B** Quantitative analysis of erythroid-associated and globin proteins by densitometry after normalization to the loading control is shown. Data are mean ± SD (*n* = 3). ^*^P < 0.05 versus pLenti cells on the same day of culture; two-sided Student's *t* test. **C** qPCR of genes encoding key proteins in **A**. Data were normalized to *GAPDH* and represented in a heatmap

Among the tested erythroid regulators, we observed a striking difference in BCL11A level in OGAi and OGTi cells when compared to pLenti cells, particularly at the later stage of differentiation on days 7 and 10 (Fig. 6A and B). The level of BCL11A was found to increase or decrease markedly with the level of *O*-GlcNAcylation, suggesting that BCL11A is a potential target protein of *O*-GlcNAcylation herein. qPCR analysis showed that *BCL11A* transcription was not changed in correspondence with its protein or the *O*-GlcNAc level (Fig. 6C), thus confirming that BCL11A protein is regulated at a posttranslational level. In humans, there is a dynamic regulation of GATA1 and GATA2 activity during erythropoiesis. When GATA1 is activated in the CFU-E stage onward, GATA2 is repressed. After which, GATA1 declines at the very last stages of terminal erythroid differentiation, likely via a caspase-dependent mechanism [29, 35]. We observed a relatively low GATA2 level at the later stages of erythroid differentiation in all cells; however, the GATA1 level fluctuated, likely due to the mixed population of late-stage erythroblasts obtained. Neither GATA level correlated well with the *O*-GlcNAcylation. Interestingly, we observed a remarkable increase in β-globin and, to a lesser extent, α-globin production in OGAi K562 cells when compared to pLenti cells even before the start of erythroid differentiation (day −1) and upon erythroid differentiation at various times (days 4−10), indicating that cellular *O*-GlcNAcylation also affected globin production.

### Inhibition of OGA mediates erythroid differentiation and globin production via BCL11A

**Fig. 7:**
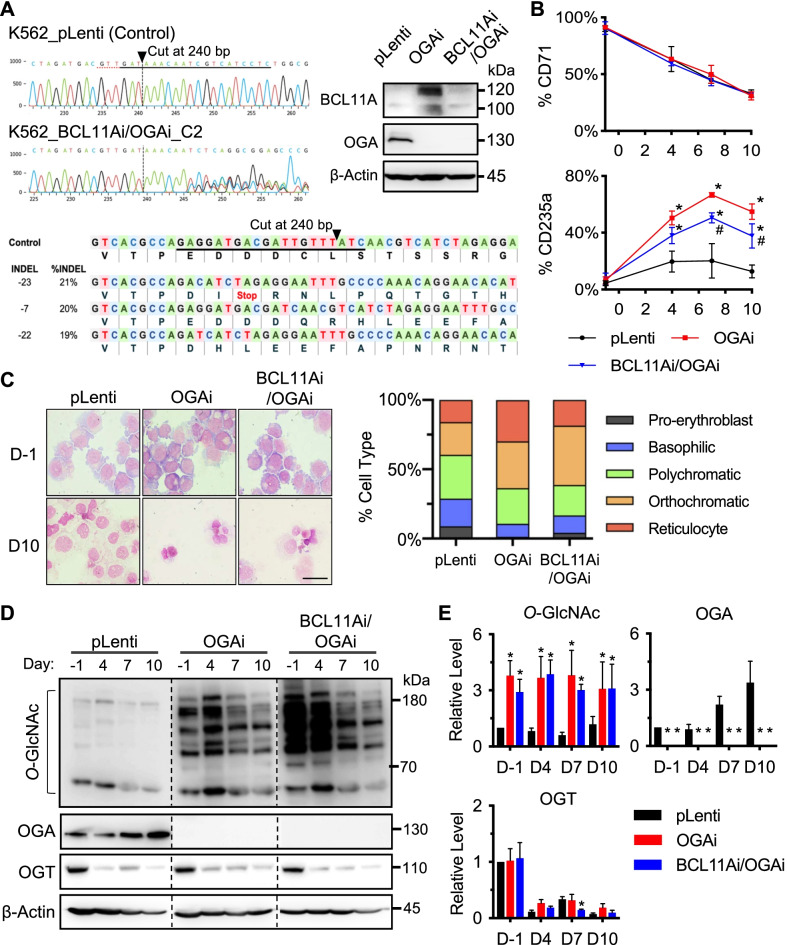
BCL11A is a key mediator of *O*-GlcNAc-mediated erythroid differentiation in K562 cells. **A** (left) DNA sequencing spanning over the Cas9 cut site on *BCL11A* from the CRISPR control (pLenti) cells in comparison with the double knockdown BCL11Ai/OGAi cells. The guide sequence is underlined in black, and the PAM sequence is denoted by a dotted red underline in the control sample. Vertical dotted lines denote the expected cut site. (right) Western blot analysis of BCL11A and OGA levels showing knockdown efficiency in BCL11Ai/OGAi cells in comparison with the single knockdown OGAi cells. (lower) The inferred sequences of *BCL11A* present in BCL11Ai/OGAi cells and their relative proportions (%INDEL) were listed along with the translated amino acids. **B** Percentages of CD71^+^ and CD235a^+^ cells at various times of culture in the optimized EPO-based medium. Data are mean ± SD (*n* = 3). ^*^P < 0.05 versus pLenti cells on the same day of culture; one-way ANOVA with Tukey’s posttest. ^#^*P* < 0.05 versus OGAi cells on the same day of culture; one-way ANOVA with Tukey’s posttest. **C** Representative morphology of differentiated cells on Wright-stained cytospin slides before imatinib pre-exposure (day −1) and after 10 days of culture (left) and its scoring for percentages of cells in different stages (right). Scale bar = 30 μm. **D** Western blot analysis of cellular *O*-GlcNAc, OGA, and OGT levels upon terminal erythroid differentiation at various times (day −1 to day 10). Blots were reprobed with anti-β-actin antibody to establish a loading control. The dashed lines separate juxtapose lanes taken from the same experiment and the same blot image. **E** Quantitative analysis of *O*-GlcNAc, OGA, and OGT levels by densitometry after normalization to the loading control is shown. Data are mean ± SD (*n* = 3). ^*^P < 0.05 versus pLenti cells on the same day of culture; one-way ANOVA with Tukey’s posttest

**Fig. 8:**
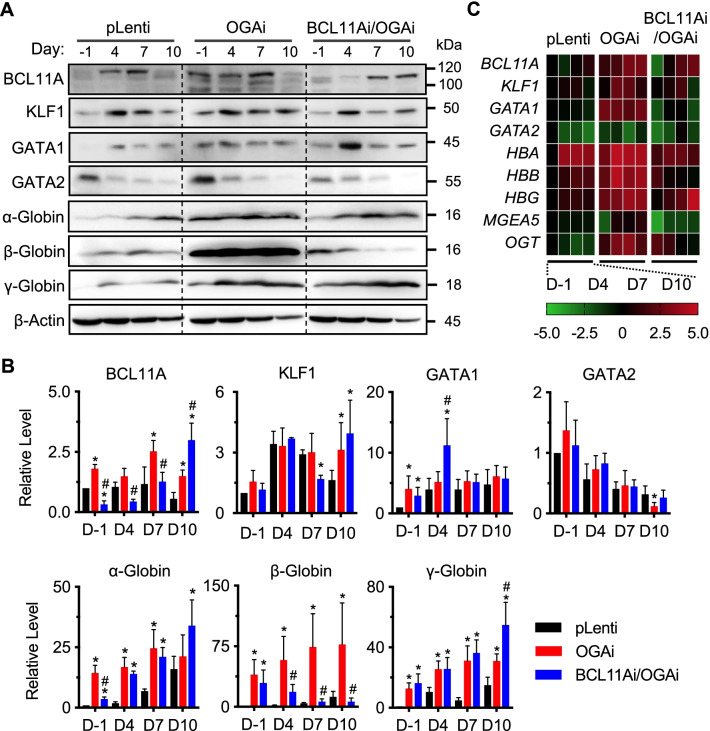
Effects of BCL11A inhibition on the *O*-GlcNAc-mediated erythroid-associated and globin proteins in K562 cells. **A** Western blot analysis of erythroid-associated proteins, including BCL11A, KLF1, GAT1, and GATA2, and globin proteins, including α-, β-, and Ɣ-globin, in OGAi, BCL11Ai/OGAi, and control (pLenti) cells upon erythroid differentiation in EPO-based medium at various times (day −1 to day 10). Blots were reprobed with anti-β-actin antibody to establish a loading control. The dashed lines separate juxtapose lanes taken from the same experiment and the same blot image. **B** Quantitative analysis of erythroid-associated and globin proteins by densitometry after normalization to the loading control is shown. Data are mean ± SD (*n* = 3 or 4). ^*^P < 0.05 versus pLenti cells on the same day of culture; one-way ANOVA with Tukey’s posttest. ^#^*P* < 0.05 versus OGAi cells on the same day of culture; one-way ANOVA with Tukey’s posttest. **C** qPCR of genes encoding key proteins in **A**. Data were normalized to *GAPDH* and represented in a heatmap

We hypothesized that hyper-*O*-GlcNAcylation via OGA inhibition enhances erythroid differentiation via BCL11A. To test this possibility, we used the CRISPR/Cas9 system to repress *BCL11A* in OGAi cells to create BCL11Ai/OGAi double knockdown cells and assessed its erythroid differentiation in comparison with OGAi cells. Figure 7A shows the efficient gene editing (71% ICE editing efficiency) and the confirmed decrease in BCL11A level in BCL11Ai/OGAi cells when compared to its counterpart control OGAi cells. Upon erythroid differentiation, the percentage of cells expressing early erythroid marker CD71 appeared to be similar between BCL11Ai/OGAi cells and OGAi cells at all time points (Fig. 7B). A lower percentage of cells expressing late erythroid marker CD235a was observed in BCL11Ai/OGAi cells than in OGAi cells at 7 and 10 days after differentiation, although it was still higher than that of CRISPR control (pLenti) cells. Morphological appearances of BCL11Ai/OGAi cells by Wright staining exhibited greater orthochromatic erythroblasts and fewer reticulocytes when compared to OGAi cells (Fig. 7C), thus supporting the immunophenotypic data and indicating that *O*-GlcNAcylation regulates erythroid differentiation in part via BCL11A. The persistent hyper-*O*-GlcNAcylation in BCL11Ai/OGAi cells strengthens that BCL11A acts downstream of *O*-GlcNAcylation (Fig. 7D and E). Notably, the protein levels of other erythroid regulators, including KLF1, GATA1, and GATA2, were similar in BCL11Ai/OGAi cells and OGAi cells (Fig. 8A and B).

Having demonstrated that OGA inhibition caused the huge increase in β-globin level, we evaluated whether inhibition of BCL11A in OGAi cells would reverse such effect. Figure 8B shows that β-globin was indeed decreased in BCL11Ai/OGAi cells when compared to its counterpart control OGAi cells after erythroid differentiation for 4, 7, and 10 days, although the basal β-globin level before the start of differentiation (day −1) was not different. In contrast, a decrease in α-globin level was observed at the basal stage but not after differentiation when compared between BCL11Ai/OGAi and OGAi cells. These data indicate that OGA inhibition and subsequent hyper-*O*-GlcNAcylation regulates β- and α-globin production, although at different stages of cells, via BCL11A.

### Inhibition of OGA generates unique FTIR signatures upon erythroid differentiation

**Fig. 9:**
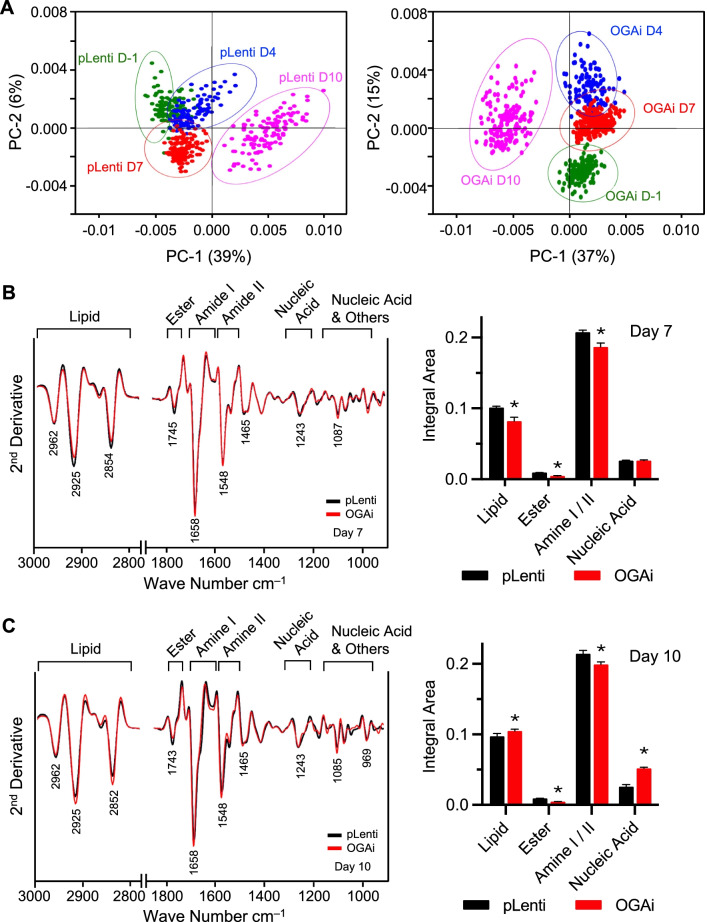
FTIR analysis upon *O*-GlcNAc-mediated erythroid differentiation in K562 cells. FTIR spectra were recorded from more than 200 single cells in the mid-IR region of 4000 to 800 cm^−1^. **A** Two-dimensional PCA score plots of control (pLenti) (left) and OGAi (right) cells upon erythroid differentiation in the EPO-based medium at various times (day −1 to day 10) showing distinct patterns. **B**, **C** (left) The second derivative spectra obtained from the mean FTIR spectra of pLenti and OGAi cells in the wavelength range of 3000−2800 cm^−1^ and 1750−800 cm^−1^. Band assignments for the region of lipid, ester (lipid), amide I, amide II, and nucleic acid, glycoprotein, and other carbohydrate (nucleic acid and others) on days 7 (**B**) and 10 (**C**) of differentiation were illustrated. (right) Integral area of total lipid, ester (lipid), amide I and amide II, nucleic acid (DNA/RNA) and nucleic acid and others on days 7 (**B**) and 10 (**C**) were plotted. Data are mean ± SD (*n* = 3). ^*^P < 0.05 versus *p*Lenti cells on the same day of culture; two-sided Student's *t* test

FTIR spectroscopy is a sensitive technique capable of characterizing and describing the biochemical contents of macromolecules in cells and tissues, which could be linked to the stage of differentiation and the physiological state of cells [21, 36, 37]. To confirm that OGA inhibition affected erythroid differentiation, differentiated cells derived from OGAi and CRISPR control (pLenti) K652 cells were collected at various times after culture and subjected to FTIR analysis. FTIR spectra were recorded from more than 200 single cells in the mid-IR region of 4000 to 800 cm^−1^. Principal component analysis (PCA) of spectroscopic data from OGAi and pLenti cells suggested distinct differentiation patterns between them (Fig. 9A). To gain more insight into their differences, various biochemical contents of OGAi and pLenti cells at 7 and 10 days after differentiation were compared using integral areas of specified FTIR spectral regions corresponding to C–H from lipid (3000–2800 cm^−1^), C=O ester primarily from lipid (1750–1735 cm^−1^), amide I protein (1670–1600 cm^−1^), amide II protein (1560–1500 cm^−1^), P=O phosphodiester bond from nucleic acid (1260–1200 cm^−1^), C–C from nucleic acid and C–O from glycoprotein and other carbohydrate (nucleic acid and others; 1180–990 cm^−1^) and RNA (975–950 cm^−1^), as illustrated in Fig. 9B and C. Significant differences in lipid, ester from lipid, and amide II protein between OGAi and pLenti cells were observed at both days. Interestingly, a striking increase in the contents of nucleic acid, glycoprotein, which could be *O*-GlcNAcylated protein, and/or carbohydrate, was found in OGAi cells after 10 days of differentiation. Altogether, these data supported the unique metabolic reprogramming in OGAi cells that could in part affect erythroid differentiation.

## Discussion

Large-scale, in vitro production of reticulocytes and other blood products from renewable cell sources has gained increasing attention, due to the limited donor availability, the potential risk of alloimmunization and other transfusion-related complications. However, the clinical application of cultured reticulocytes faces several constraints, such as the limited proliferation and inability to generate a large cell number, a low enucleation rate, and the expression of fetal rather than adult hemoglobin, especially when the starting cells are ESCs and iPSCs [4–6, 8, 9]. These issues might be overcome by a better understanding of the mechanisms that regulate erythropoiesis and globin production.

During human hematopoiesis, both erythrocytes and megakaryocytes are derived from a committed progenitor, called megakaryocyte–erythroid progenitors (MEPs) [38, 39]. We have demonstrated in our recent study that metabolic sensor *O*-GlcNAcylation plays an essential role in megakaryopoiesis from HSPCs and platelet production from megakaryoblasts/megakaryocytes via the regulation of c-Myc-mediated integrin-α4 and integrin-β7 [40]. In this study, we extend the knowledge on the roles of *O*-GlcNAcylation in erythropoiesis, starting from the lineage specification of HSPCs to terminal differentiation and erythroid maturation. We reveal that two-step inhibition of OGT and OGA (OGTinh/OGAinh) improves the lineage specification and differentiation of HSPCs toward matured erythroblasts (Fig. 2). The hint about this treatment strategy came from the dynamic change in the cellular *O*-GlcNAc level during erythropoiesis, which was initially low in HSPCs, then gradually increased in the early phase and decreased in the last stage of differentiation (Fig. 1D). Notably, the promoting role of OGT inhibition in erythroid lineage specification in the early phase of erythropoiesis was supported by the finding that OGTinh OSMI-1 steered the HSPC-derived CFUs toward erythroid progenitor BFU-E (Fig. 1A). In the efficient K562 model of erythroid differentiation, which started at the erythroblastic stage, inhibition of OGA and subsequent increase in *O*-GlcNAcylation induce erythroid maturation, enucleation, and globin production—both -globin and, to a lesser extent, α-globin.

*O*-GlcNAcylation is a posttranslational modification that regulates nearly all aspects of cellular processes through changes in protein function, gene expression, and cellular signaling [13, 41, 42]. Thus far, more than 4,000 proteins have been identified as targets of *O*-GlcNAcylation [43]. BCL11A is a zinc finger transcription factor that has been shown to repress Ɣ-globin expression and fetal hemoglobin in human erythroid cells [31]. The robust expression of the full-length form of BCL11A is developmentally restricted to adult erythroid cells—it expressed at substantially lower levels in fetal cells and absent in primitive erythroblasts [44, 45]. We found that BCL11A is a key mediator of *O*-GlcNAc-driven erythroid differentiation and globin production herein. Once BCL11A was downregulated in OGAi cells, the promoting effect of OGA inhibition on erythroid cells was relieved (Fig. 8). Consistent with previous studies, we also observed the increased Ɣ-globin expression upon knockdown of BCL11A. In humans, *BCL11A* expression is in part controlled by KLF1, the erythroid master regulator that directly activates β-globin gene expression and indirectly represses -globin [46]. During fetal development, it appeared that low level of KLF1 was insufficient to activate *BCL11A* and subsequent Ɣ-globin repression. In adult erythroid progenitors, increased level of KLF1 resulted in the formation of complexes that provided β-globin gene with a competitive advantage for interaction with the locus control region (LCR) and activated *BCL11A*, which inhibited LCR-Ɣ-globin gene interactions. We provide evidence that BCL11A is posttranslationally controlled by OGA inhibition and hyper-*O*-GlcNAcylation and that BCL11A regulates β-globin level, although through an unknown mechanism. Additionally, α-globin has been affected by this *O*-GlcNAc/BCL11A regulatory axis.

FTIR spectroscopy has been applied in the biomedical field to study global structural and compositional changes in various cells and tissues. With the high signal-to-noise ratio of less than 10 μm per spot on the sample and enhanced diffraction-limited lateral spatial resolution, it can be used to identify even a small difference in biochemical contents at the single-cell level [47]. Our FTIR data showed unique metabolic fingerprints upon OGA inhibition during erythroid differentiation that support the involvement of metabolic reprogramming, i.e., lipid, nucleic acid, and glycoprotein/carbohydrate, in the process. It is worth noting that our studies do not exclude roles for additional factors in erythropoiesis and globin production, which requires further investigation. We merely emphasize the roles of *O*-GlcNAcylation via BCL11A, which are the focus of our present investigations.

## Conclusions

In summary, the evidence presented here demonstrates the significance of the *O*-GlcNAc/BCL11A axis in regulating erythropoiesis and globin production. We believe that the novel insight gained from our study could be important in understanding hematologic disorders whose etiology is related to impaired erythroid differentiation and maturation, and hemoglobinopathies. Our findings may lay groundwork for future clinical applications toward an ex vivo production of human reticulocytes for transfusion from renewable cell sources.

## Supplementary Information

**Additional file 1: Table S1**. Key resources table. **Table S2**. The oligo sequences of sgRNAs and plasmid information. **Table S3**. List of primers used for qPCR. **Table S4**. Percentages of multiple types of progenitor cell colonies in the CFU assay of UCB-derived CD34^+^ HSPCs. **Table S5**. Characteristics of UCB-derived HSPC donors.

**Additional file 2: Fig. S1.** Representative images of plates and micrographs of different progenitor cell colonies from CFU assay, in correspond to the data in Fig. 1A. **Fig. S2.** Gene and protein profiles of *O*-GlcNAc cycling enzymes during erythroid differentiation in EPO-based medium. **Fig. S3.** Immunophenotypic profile of differentiated cells in EPO-based medium. **Fig. S4. **Flow cytometry gating strategy using CD71 and FSC together with CD235a to distinguish erythroid cells into different subsets. **Fig. S5.** Percentages of differentiated erythroblasts in stages I−V as analyzed by flow cytometry using FSC versus CD71. **Fig. S6. **UBC-derived CD34^+^ HSPCs were treated with either OGAinh/OGTinh or OGTinh/OGAinh, switching on day 11, for a total of 15 days. **Fig. S7.** Cell viability of erythroid cells derived from human-derived erythroblastic K562 cells using Protocol 0−4 on day 10 of culture. **Fig. S8.** Images of K562 cell pellets obtained before imatinib pre-exposure (day −1) and after 4, 7, and 10 days of culture in EPO-based medium for erythroid differentiation.

## Data Availability

The datasets generated and/or analyzed during the current study are available from the corresponding author on reasonable request. DNA sequencing data are available at GenBank (https://www.ncbi.nlm.nih.gov/genbank/) under accession numbers: ON645646 (*MGEA5* in pLenti), ON645647 (*MGEA5* in OGAi), ON645648 (*OGT* in pLenti), ON645649 (*OGT* in OGTi), ON645650 (*BCL11A* in pLenti), and ON645651 (*BCL11A* in BCL11Ai/OGAi). Supplementary information is available.

## Abbreviations

ATCC: American Type Culture Collection
BCL11Ai/OGAi: BCL11A and OGA double knockdown cells
BFU-E: Burst-forming unit-erythroid
BSA: Bovine serum albumin
CFU: Colony-forming unit
CFU-E: CFU-erythroid
CFU-GEMM: CFU-granulocyte, erythrocyte, macrophage, megakaryocyte
CFU-GM: CFU-granulocyte, macrophage
EPO: Erythropoietin
FACS: Fluorescence-activated cell sorting
FBS: Fetal bovine serum
FSC: Forward scatter
FTIR: Fourier transform infrared
GlcNAc: N-acetylglucosamine
HBP: Hexosamine biosynthetic pathway
hESC: Human embryonic stem cell
hiPSC: Human-induced pluripotent stem cell
HSC: Hematopoietic stem cell
HSPC: Hematopoietic stem/progenitor cell
ICE: Inference of CRISPR Edits
IMDM: Iscove’s modified Dulbecco’s medium
INDEL: Insertion/deletion
LPR: Locus control region
MC: Methylcellulose
MEP: Megakaryocyte–erythroid progenitor
NaB: Sodium butyrate
OGA: *O*-GlcNAcase
OGAinh: OGA inhibitor
OGT: *O*-GlcNAc transferase
OGTi: OGT knockdown cells
OGTinh: OGT inhibitor
PCA: Principal component analysis
qPCR: Quantitative real-time PCR
rh: Recombinant human
sgRNA: Single-guide RNA
SMI: Small molecule inhibitor
UCB: Umbilical cord blood
UTP: Nucleotide uridine-5’-triphosphate
