# Correction: Metabolic sensor *O*-GlcNAcylation regulates erythroid differentiation and globin production via BCL11A

**DOI:** 10.1186/s13287-022-03098-2

**Published:** 2022-08-03

**Authors:** Sudjit Luanpitpong, Xing Kang, Montira Janan, Kanjana Thumanu, Jingting Li, Pakpoom Kheolamai, Surapol Issaragrisil

**Affiliations:** 1 Siriraj Center of Excellence for Stem Cell Research, Faculty of Medicine Siriraj Hospital, Mahidol University, 2 Siriraj Hospital, Bangkoknoi, Bangkok, 10700 Thailand; 2 Synchrotron Light Research Institute (Public Organization), Nakhon Ratchasima, Thailand; 3 Institute of Precision Medicine, Department of Burns, The First Affiliated Hospital, Sun Yat-Sen University, Guangzhou, China; 4 Center of Excellence in Stem Cell Research and Innovation, Faculty of Medicine, Thammasat University, Pathum Thani, 12120 Thailand; 5 Division of Hematology, Department of Medicine, Faculty of Medicine Siriraj Hospital, Mahidol University, Bangkok, Thailand

## Correction to: Stem Cell Research & Therapy  (2022) 13, 274 10.1186/s13287-022-02954-5

Following publication of the original article [1], the authors identified inadvertent errors during manuscript preparation that incorrect labels have been placed in Fig. 9. They labeled the functional group ‘Amide I’ and ‘Amide II’ in Fig. 9B and C as ‘Amine I’ and ‘Amine II’ by mistake. The label ‘Nucleic Acid & Others’ of the bar plots was labeled as ‘Nucleic acid’ due to errors in typesetting.

**Fig. 9:**
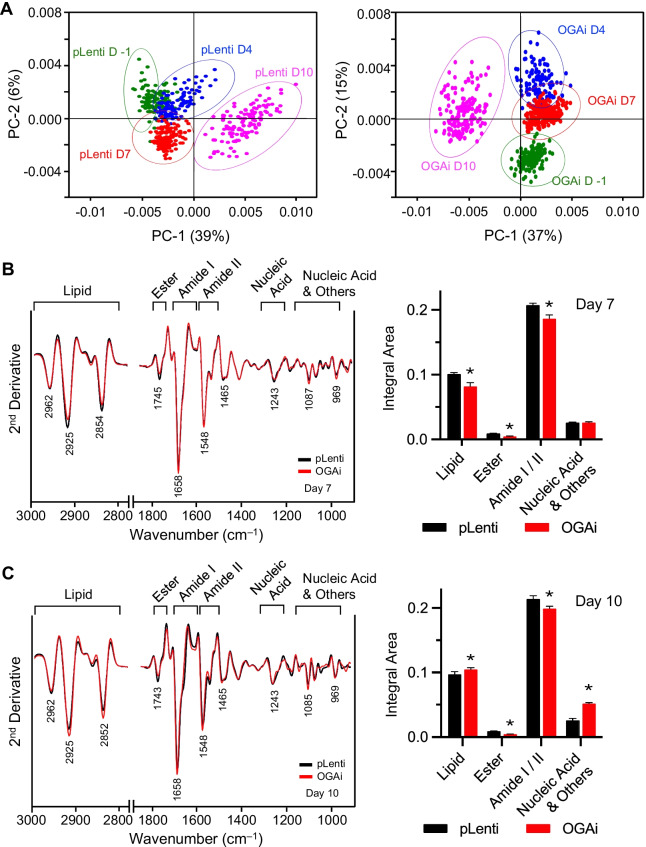
FTIR analysis upon *O*-GlcNAc-mediated erythroid differentiation in K562 cells. FTIR spectra were recorded from more than 200 single cells in the mid-IR region of 4000 to 800 cm^−1^. **A** Two-dimensional PCA score plots of control (pLenti) (left) and OGAi (right) cells upon erythroid differentiation in the EPO-based medium at various times (day − 1 to day 10) showing distinct patterns. **B**, **C** (left) The second derivative spectra obtained from the mean FTIR spectra of pLenti and OGAi cells in the wavelength range of 3000–2800 cm^−1^ and 1750–800 cm^−1^. Band assignments for the region of lipid, ester (lipid), amide I, amide II, nucleic acid (DNA/RNA), and nucleic acid, glycoprotein, other carbohydrate (nucleic acid and others) on days 7 (**B**) and 10 (**C**) of differentiation were illustrated. (right) Integral area of total lipid, ester (lipid), amide I and amide II, and nucleic acid and others on days 7 (**B**) and 10 (**C**) were plotted. Data are mean ± SD (n = 3). ^*^P < 0.05 versus pLenti cells on the same day of culture; two-sided Student's *t* test

The corrected figure is given in this article.
