# Supplementary material for: Metabolic sensor O-GlcNAcylation regulates erythroid differentiation and globin production via BCL11A
Source: Stem Cell Res Ther. 2022 Jun 23;13:274. doi: 10.1186/s13287-022-02954-5 (PMC9219246; doi:10.1186/s13287-022-02954-5)
Supplement: Supplementary file 1 — Additional file 1: Table S1. Key resources table. Table S2. The oligo sequences of sgRNAs and plasmid information. Table S3. List of primers used for qPCR. Table S4. Percentages of multiple types of progenitor cell colonies in the CFU assay of UCB-derived CD34+ HSPCs. Table S5. Characteristics of UCB-derived HSPC donors. [file 13287_2022_2954_MOESM1_ESM.pdf]

SUPPLEMENTARY TABLES

Table S1. Key resources table.

**Reagents**	**Source**	**Catalog number**
Chemicals and inhibitors		
Alloxan	Sigma-Aldrich	A7413
Decitabine (5-Aza-2′-deoxycytidine)	Sigma-Aldrich	A3656-
Dimethyl sulfoxide (DMSO)	Merck	102952
Imatinib	Sigma-Aldrich	Y0001694
NaB	Sigma-Aldrich	B5887
OSMI-1	Sigma-Aldrich	SML1621
Rapamycin	Sigma-Aldrich	R0395
Thiamet G	Tocris Bioscience	4390
TRIzol reagent	Invitrogen	1IV11-15596-026
Media, cytokines, and recombinant proteins		
Bovine serum albumin (BSA)	Sigma-Aldrich	A7906
Fetal bovine serum (FBS)	PAN-Biotech	P30-3033
Ficoll-Paque PLUS	GE Healthcare	95038
Heparin LEO	LEO Pharmaceutical	N/A
Insulin solution human	Sigma-Aldrich	I9278
IMDM, powder	Thermo Fisher Scientific	12200-036
MethoCult™ H4435 Enriched	STEMCELL Technologies	04435
rhEPO (EPREX®)	Janssen-Cilag	N/A
rhFLT3-L, research grade	Miltenyi	130-096-474
rhIL-3, research grade	Miltenyi	130-093-909
rhIL-6, research grade	Miltenyi	130-095-365
rhSCF, premium grade	Miltenyi	130-093-864
holo-Transferrin human	Sigma-Aldrich	T0665
Antibodies, cocktails, and kits		
Anti-β-Actin (8H10D10)	Cell signaling Technology	3700
Anti-hemoglobin α (D-4)	Santa Cruz Biotechnology	sc-514378
Anti-hemoglobin β (37-8)	Santa Cruz Biotechnology	sc-21757
Anti-hemoglobin ɣ (51-7)	Santa Cruz Biotechnology	sc-21756
Anti-BCL11A (14B5)	Abcam	ab19487
Anti-KLF1	Abcam	ab56011
Anti-GATA1	Abcam	ab28839
Anti-GATA2	Abcam	ab153820
Anti-O-GlcNAc (O-Linked N-Acetyl-glucosamine) (RL2)	Abcam	ab2739

Anti-MGEA5/OGA (EPR7154(B))	Abcam	ab124807
Anti-OGT / O-Linked N-Acetyl-glucosamine Transferase	Abcam	ab96718
APC anti-human CD235a	BioLegend	349114
CD34 MicroBead Kit	Miltenyi	130-046-702
cOmplete™ Protease Inhibitor Cocktails	Roche Diagnostics	11697498001
FITC anti-human CD71	BioLegend	334104
Cell Lysis Buffer (10X)	Cell Signaling Technology	9803
MS Columns	Miltenyi	130-042-201
PerCP-Cy5.5 anti-human CD235a	BioLegend	349110
Power SYBR™ Green PCR Master mix	Applied Biosystems	4367659
RevertAid First Strand cDNA Synthesis Kit	Thermo Fisher Scientific	K1622

Table S2. The oligo sequences of sgRNAs and plasmid information.

Name	sgRNA target sequence (5'−3')	Vector / Delivery method	Vector type	Selection marker
OGA CRISPR Guide 3	GTCCAATTTACGTTTCAATG	pLentiCRISPR v2 / Lentiviral	All-in-one (SpCas9 & sgRNA)	AmpR, PuroR
OGT CRISPR Guide 1	AGCCAATATCAAACGAGAAC	pLentiCRISPR v2 / Lentiviral	All-in-one (SpCas9 & sgRNA)	AmpR, PuroR
BCL11A CRISPR Guide 1	GATAAACAATCGTCATCCTC	pLentiCRISPR v2 / Lentiviral	All-in-one (SpCas9 & sgRNA)	AmpR, PuroR

Table S3. List of primers used for qPCR.

Target	Forward / Reverse primer (5'−3')
TAL1	CTATGAGATGGAGATTACTGATGGTC / GTGTGGGGATCAGCTTGC
GATA2	TTGTGCAAATTGTCAGACGAC / TCATGGTCAGTGGCCTGTTA
RUNX1	CTCCCTGAACCACTCCACTG / TGGGGATGGTTGGATCTG
GATA1	CACTGAGCTTGCCACATCC / ATGGAGCCTCTGGGGATTA
KLF1	ACACCAAGAGCTCCCACCT / GTAGTGGCGGGTCAGCTC
BCL11A	CCAAACAGGAACACATAGCAGA / GAGCTCCATGTGCAGAACG
HBA	GACCCGGTCAACTTCAAGC / AGAAGCCAGGAACTTGTCCA
HBG	TGGATCCTGAGAACTTCAAGC / GCCACTGCAGTCACCATCT
HBB	GCACGTGGATCCTGAGAACT / CACTGGTGGGGTGAATTCTT
HBE	GGTGAAGCCTTGGGCAGACTCC / TGCCATGGGCCTTGACCTTG
OGT	GCAGCAGGACCAATTACCTC / GCATACGTTTCGTTGGTTCTG
MGEA5	TGGTCTAGCAGGAGAGTTCCA / AAACTTTGGAGGTAGGAGTCAGTG
GADPH	AGCCACATCGCTCAGACAC / GCCCAATACGACCAAATCC

Table S4. Percentages of multiple types of progenitor cell colonies in the CFU assay of UCB-derived $CD34^+$ HSPCs.

Treatment	% CFU-GM	% CFU-GEMM	% CFU-E	% BFU-E
Control (CTL)	79.43 ± 20.92	12.68 ± 15.66	1.69 ± 2.30	6.20 ± 4.85
Thiamet G (TMG)	79.24 ± 12.76	11.36 ± 9.09	0.75 ± 1.34	8.66 ± 3.82
OSMI-1	71.71 ± 17.58	8.86 ± 9.48	4.10 ± 4.90	15.33 ± 6.17*

Colonies were visualized and scored under an inverted microscope at 14 days of culture and percentage of each progenitor cell colony over total number of colonies was calculated. Data are means ± s.d. (n = 6) from six independent experiments. $^*P < 0.05$ versus % BFU-E of nontreated control; two-sided Student's t test.

Table S5. Characteristics of UCB-derived HSPC donors.

Designation	#7	#14	#23
Mother			
Age (years)	37	20	35
Nationality	Thai	Cambodian	Burmese
Disease presence	–	–	–
Gestational age (weeks)	38	41	38
Mode of delivery	Vaginal	Vaginal	Vaginal
Newborn			
Sex	Male	Male	Female
Birth order	1	1	2
Birth weight (g)	2,810	3,290	3,530
Umbilical cord weight (g)	470	700	700
