# Supplementary material for: Metabolic sensor O-GlcNAcylation regulates erythroid differentiation and globin production via BCL11A
Source: Stem Cell Res Ther. 2022 Jun 23;13:274. doi: 10.1186/s13287-022-02954-5 (PMC9219246; doi:10.1186/s13287-022-02954-5)
Supplement: Supplementary file 2 — Additional file 2: Fig. S1. Representative images of plates and micrographs of different progenitor cell colonies from CFU assay, in correspond to the data in Fig. 1A. Fig. S2. Gene and protein profiles of O-GlcNAc cycling enzymes during erythroid differentiation in EPO-based medium. Fig. S3. Immunophenotypic profile of differentiated cells in EPO-based medium. Fig. S4. Flow cytometry gating strategy using CD71 and FSC together with CD235a to distinguish erythroid cells into different subsets. Fig. S5. Percentages of differentiated erythroblasts in stages I−V as analyzed by flow cytometry using FSC versus CD71. Fig. S6. UBC-derived CD34+ HSPCs were treated with either OGAinh/OGTinh or OGTinh/OGAinh, switching on day 11, for a total of 15 days. Fig. S7. Cell viability of erythroid cells derived from human-derived erythroblastic K562 cells using Protocol 0−4 on day 10 of culture. Fig. S8. Images of K562 cell pellets obtained before imatinib pre-exposure (day −1) and after 4, 7, and 10 days of culture in EPO-based medium for erythroid differentiation. [file 13287_2022_2954_MOESM2_ESM.pdf]

**SUPPLEMENTARY FIGURES**

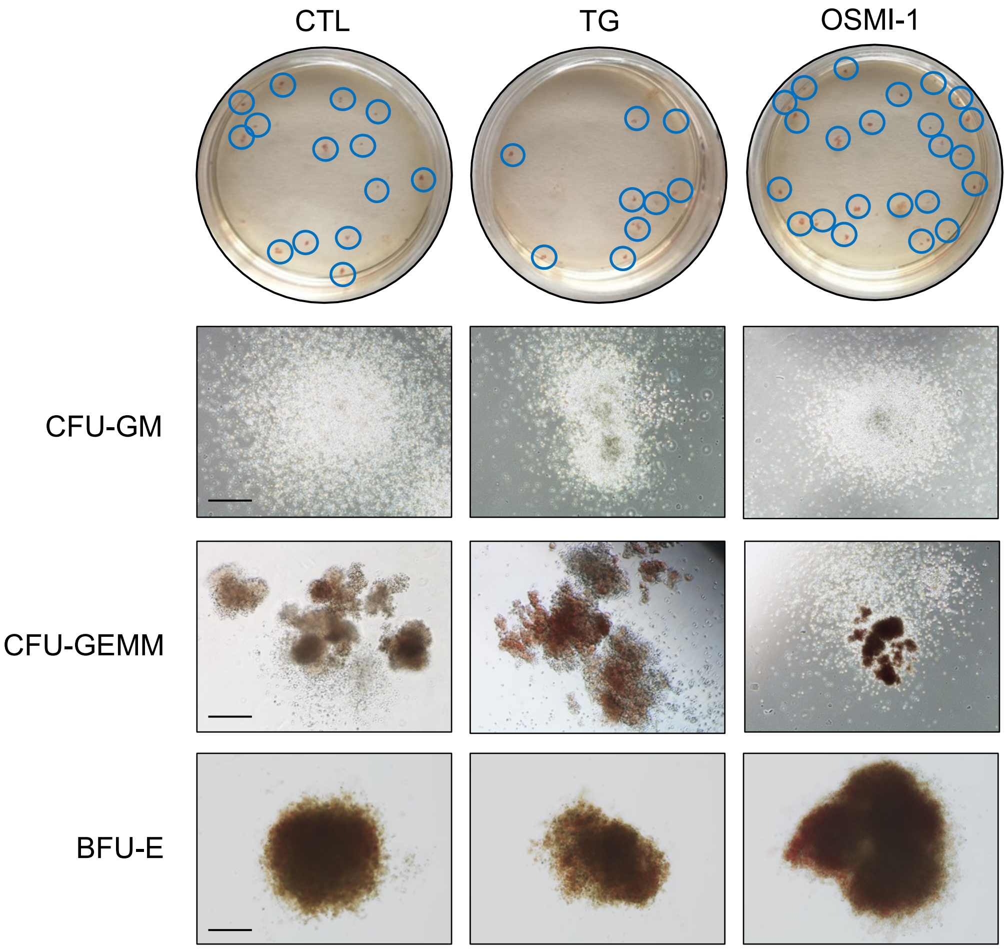


**Fig. S1.** Representative images of plates and micrographs of different progenitor cell colonies from CFU assay, in correspond to the data in Fig. 1A. Circles outline the large, visible BFU-E colonies. Scale bar = 200 μm.

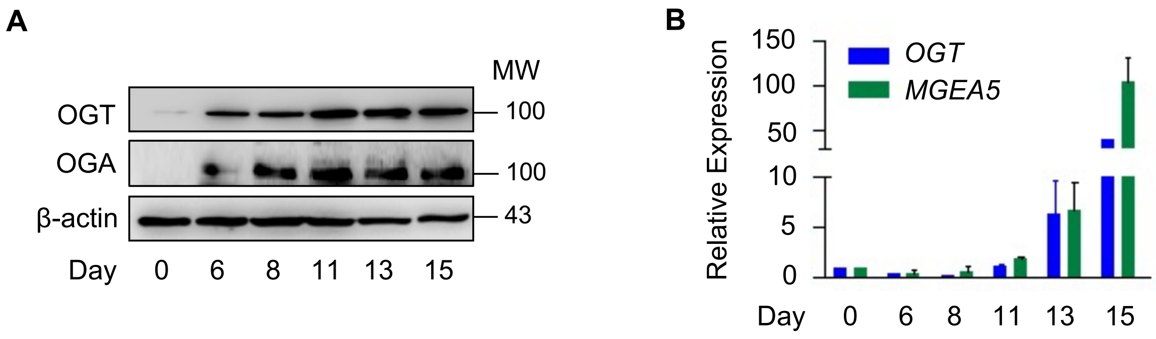


**Fig. S2.** Gene and protein profiles of *O*-GlcNAc cycling enzymes during erythroid differentiation in EPO-based medium. (A) Western blot analysis of OGT and OGA at various times of culture (day 0–day 15). Blots were reprobed with anti-β-actin antibody to establish a loading control. (B) qPCR of genes encoding OGT and OGA (*MGEA5*). Data were normalized to *GAPDH* and the *OGT* or *MGEA5* expression at the start of culture (day 0).

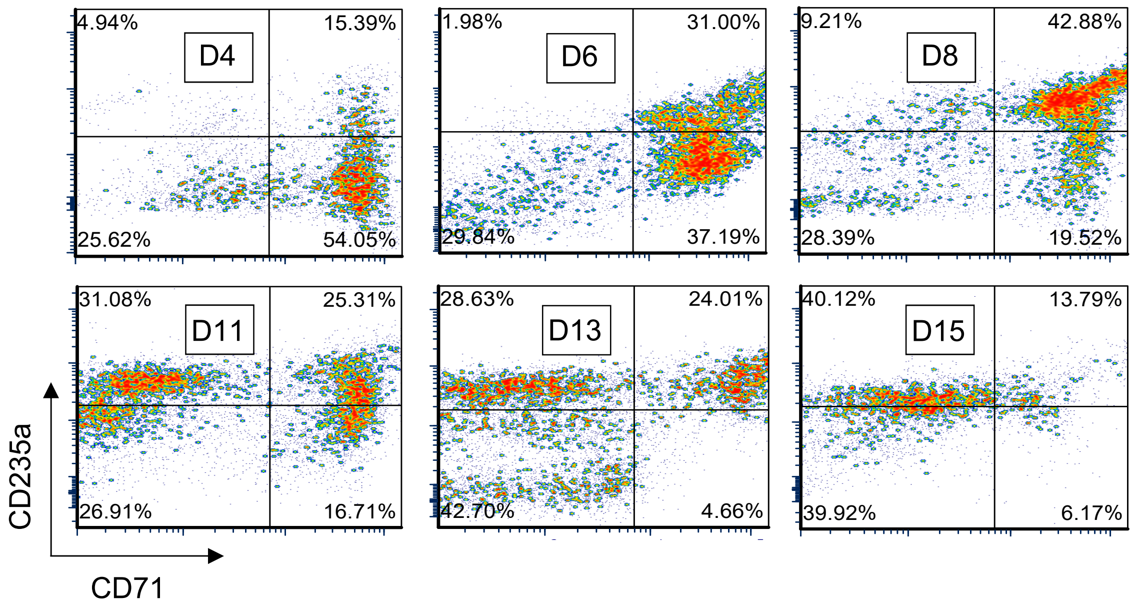


**Fig. S3.** Immunophenotypic profile of differentiated cells in EPO-based medium. Flow cytometric analysis of early erythroid marker CD71 and late erythroid marker CD235a during erythroid differentiation from UCB-derived HSPCs at various times of culture (day 0–day 15).

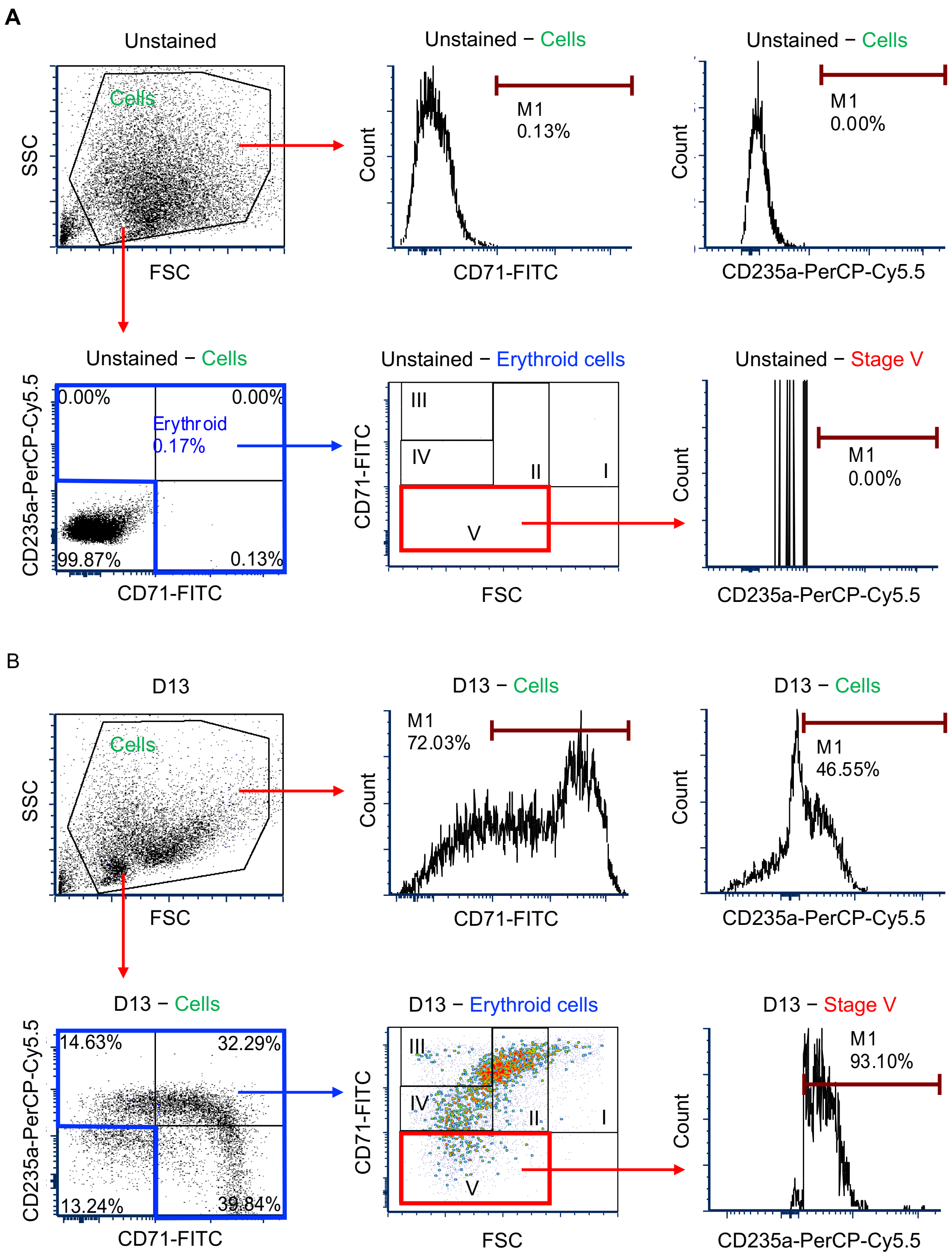


**Fig. S4.** Flow cytometry gating strategy using CD71 and FSC together with CD235a to distinguish erythroid cells into different subsets. The most mature, stage V subset was $CD71^{low}FSC^{low}CD235a^{high}$.

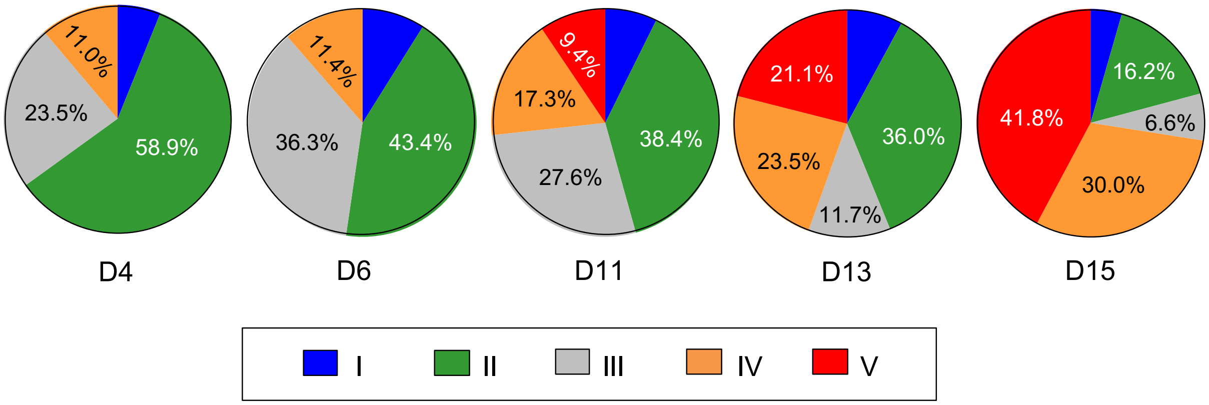


**Fig. S5.** Percentages of differentiated erythroblasts in stages I−V as analyzed by flow cytometry using FSC versus CD71. Data were derived from HSPCs of donor #6 on various days of culture (days 4−15), in correspond to the data in Fig. 1E.

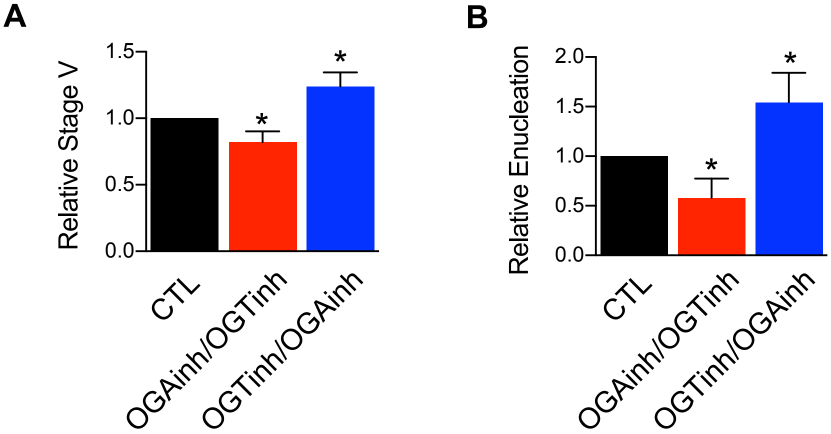


**Fig. S6.** UBC-derived $CD34^+$ HSPCs were treated with either OGAinh/OGTinh or OGTinh/OGAinh, switching on day 11, for a total of 15 days. (A, B) Percentages of differentiated erythroblasts in stages V (A) and enucleation (B) were analyzed on day 15 of culture and reported as a ratio to nontreated control (CTL). Data are means ± s.d. (n = 3), in correspond to the data in Fig. 2B and C. *$P < 0.05$ versus CTL; two-sided Student's $t$ test.

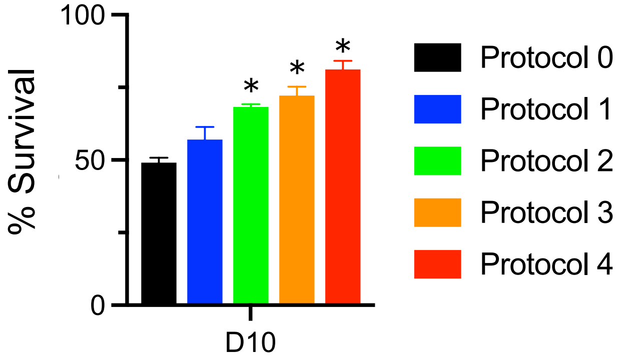


**Fig. S7.** Cell viability of erythroid cells derived from human-derived erythroblastic K562 cells using Protocol 0–4 on day 10 of culture. Data are means ± s.d. (n = 3). **P* < 0.05 versus Protocol 0; two-sided Student's *t* test.

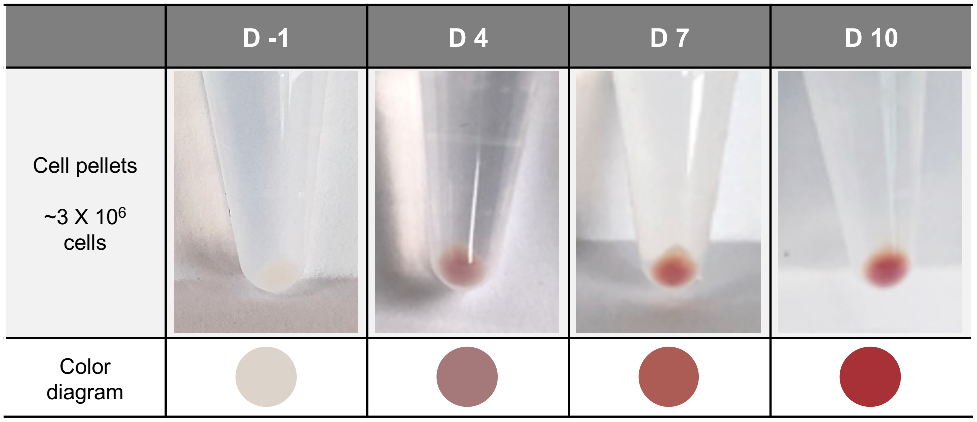


**Fig. S8.** Images of K562 cell pellets obtained before imatinib pre-exposure (day −1) and after 4, 7, and 10 days of culture in EPO-based medium for erythroid differentiation.
